# Unified Semantic Reasoning and Planning for Autonomous Driving

**DOI:** 10.3390/s26185973

**Published:** 2026-09-21

**Authors:** Ye-Chan An, Jun-Hyeon Choi, Jeong-Won Pyo, Sang-Hyeon Bae, Tae-Yong Kuc

**Affiliations:** 1 Department of Electrical and Computer Engineering, College of Information and Communication Engineering, Sungkyunkwan University, Suwon 16419, Republic of Korea; ycan@skku.edu (Y.-C.A.);; 2 DXR Co., Ltd., R&D Center, Seoul 01411, Republic of Korea; 3 Samsung Display Co., Ltd., R&D Center, Yongin 17113, Republic of Korea

**Keywords:** autonomous driving, semantic navigation, semantic reasoning, graph optimization, ontology

## Abstract

The conventional autonomy stack layers separate representations, leaving a vertical gap between high-level semantic reasoning and low-level trajectory generation. This paper closes that gap by carrying a single Triplet Ontological Semantic Model (TOSM) representation across the stack. A standardized high-definition (HD) map is first lifted into a TOSM-based semantic HD map with symbolic, explicit, and implicit attributes. Semantic Web Rule Language (SWRL) rules then infer context-appropriate maneuvers such as yielding, stopping, and lane changing. The inferred state is translated automatically into a Planning Domain Definition Language (PDDL) mission plan, and the same representation drives a Gaussian-process factor-graph optimizer that produces a smooth, kinematically feasible trajectory. The optimizer reuses the incremental factor graph of the localization layer, and semantic lane and interaction factors tie the trajectory directly to the map. Reasoning, planning, and optimization therefore share one interpretable representation, and every inferred maneuver remains traceable to the trajectory that realizes it. The rule base is logically consistent and agrees with an independent description-logic reasoner. In a closed-loop drive-along on urban routes, the optimizer tracks the routed lane far more tightly than a diverse set of baselines while remaining smooth and feasible. An open-loop evaluation on real driving data recorded in Busan, Republic of Korea, reproduces this advantage against an untuned continuous-optimization baseline.

## 1. Introduction

Autonomous vehicles on complex urban roads must reason not only about geometry but about the semantic meaning of the surrounding elements to decide what to do. The raw streams a vehicle acquires describe where things are, but not why an element matters, how elements relate, or which maneuver the current situation calls for. A pedestrian intending to cross, a lane that inherits the speed regulation of its enclosing road segment, and the maneuver each situation implies are semantic rather than purely geometric facts. Bridging the gap between sensor-level geometric perception and human-like situational interpretation, and then acting on that interpretation, is a central requirement for safe and explainable autonomous driving.

The autonomy stack is conventionally built as a modular pipeline of perception, localization, planning, and control, each sub-problem with a mature literature [1]. This work keeps that modular, auditable paradigm rather than the opaque end-to-end alternative [2,3], and augments it with an explicit semantic layer, so that behavior decisions remain traceable while the trajectory is still produced by a principled optimization.

A persistent gap nonetheless runs vertically through this stack, between high-level semantic reasoning and low-level trajectory generation. Ontology- and rule-based methods produce interpretable behavior decisions and symbolic planners sequence them, but neither specifies how an inferred maneuver becomes a smooth, kinematically feasible trajectory. Conversely, optimization-based planners excel at trajectory generation yet typically consume undifferentiated geometric inputs rather than the output of an explicit reasoning layer. Closing this gap within a single, consistent representation is the central objective of this paper.

The key enabler is a representation expressive enough to hold both the symbolic facts that a reasoner manipulates and the geometric quantities that a trajectory optimizer consumes, so that one model can be read by the behavior layer and written directly into the trajectory. The semantic high-definition (HD) map supplies the road structure and its posted regulation, such as per-lane speed limits, while surrounding vehicles tracked at run time and the ego pose enter the same model as the states on which the interaction and boundary factors act. This representation is the Triplet Ontological Semantic Model (TOSM), developed in a sustained line of work in our group: a semantic modeling framework with an on-demand semantic database for service-robot navigation [4], hierarchical multi-robot task planning [5], on-road object-centric pose estimation [6], and context-aware vehicle localization as constraint selection on a factor graph [7], which shares authors with the present work. Across this line, the shared TOSM base has served perception, localization, and task-level allocation, but it has never been used to select on-road driving behavior or to translate that behavior into an executable trajectory. Whereas the prior localization work used the shared base to decide where the vehicle is, the present work uses the same base, solved with the same incremental factor-graph inference, to decide what the vehicle should do and how it should move. Table 1 makes this delta explicit: where each prior paper in the line served a single layer (perception, localization, or task-level allocation) from the shared base, this work adds the on-road behavior, mission, and trajectory layers on the same substrate and the same factor-graph inference. It is this vertical span across a single ontological substrate, not any one of the layers in isolation, that no prior work, in our group or elsewhere, provides.

Concretely, sensor-derived attributes, environmental elements, their relations, and the ego state are described in TOSM. Semantic Web Rule Language (SWRL) rules infer context-appropriate behaviors such as yielding, stopping, and lane changing (car-following is realized geometrically by the trajectory layer, not by a rule). A Planning Domain Definition Language (PDDL) domain turns the inferred situation into an executable mission plan. The inferred behaviors together with the semantic HD-map structure are transferred into a factor-graph local trajectory optimizer, where semantic lane, interaction, and speed-cap factors shape a smooth, feasible trajectory.

The main contributions of this paper are as follows.

- We extend the Triplet Ontological Semantic Model (TOSM) into a reasoning- and optimizer-ready semantic HD map whose single, DL-consistent identifier space is shared directly by the logic reasoner and the trajectory optimizer;
- We develop an ontology-based reasoning mechanism whose threshold-free SWRL rules infer driving maneuvers and ground each inference into both a PDDL mission plan and the continuous trajectory factors;
- We propose a semantic factor-graph trajectory optimizer that ties the local trajectory to the semantic map, surrounding vehicles, and posted regulation, unifying localization, behavior, mission, and trajectory on one substrate.

By unified, we mean a single shared TOSM representation carried across the stack, so that every layer reasons over the same individuals and every inferred maneuver is traceable to the trajectory that realizes it. We do not claim a single monolithic optimization in which all sources act on one trajectory simultaneously, and route ownership between the geometric router and the symbolic mission planner is mode-selected (Section 4.6).

The remainder of this paper is organized as follows. Section 2 reviews related work and locates the vertical gap that motivates this work. Section 3 opens with a system overview and then presents the TOSM semantic HD map, the SWRL behavior rules, and the automatically generated PDDL mission planner, and Section 4 develops the semantic factor-graph trajectory optimizer. Section 5 reports the experimental validation, and Section 6 concludes.

## 2. Related Works

The autonomy stack is conventionally decomposed into perception, localization, planning, and control, with planning further divided into global routing, behavior decision-making, and local motion planning [1]. This section reviews the research strands the proposed framework draws together: motion planning and control for autonomous driving, semantic knowledge representation and reasoning, semantic maps, symbolic behavior reasoning and mission planning, and local trajectory optimization. Each is surveyed in turn, with emphasis on the gap that a unified TOSM-based approach addresses.

### 2.1. Planning and Control for Autonomous Driving

Global routing computes a lane-level path over the road network with classical graph search such as Dijkstra’s algorithm [8] or the A* heuristic [9], while local planning generates an executable trajectory for a concrete maneuver, using sampling-based [10,11], search-based [12], or optimization-based [1] methods, and a controller such as model predictive control tracks the result [13]. Complementary lines of work harden the low-level estimation and control loop itself under uncertainty and adversarial threats, for example secure observer-based collision-free control under non-Gaussian noise and data-falsification attacks [14]. These robustify the loop beneath the planner, whereas the present work addresses the representational gap above that loop, and the two are naturally combined in a full system. In reaction to the brittleness of hand-designed interfaces between these stages, end-to-end learning maps raw sensor input directly to control or to a planned trajectory [2], and recent architectures jointly optimize perception, prediction, and planning within one differentiable network [3,15], as consolidated in a recent survey of the field [16]. These learned pipelines achieve strong empirical performance but their decision process is opaque, which undermines the interpretability and verifiability increasingly required for safety accountability. The modular pipeline preserves interpretability but keeps behavior decision-making and motion generation in separate representations, so a semantic decision reaches the optimizer only through a narrow, predefined interface and never enters the optimization itself. It is this interface, not the choice of modular versus learned pipeline, that the present work targets by carrying the decision continuously from the map into the optimizer; the remaining subsections locate where prior work along each thread leaves that gap open.

### 2.2. Semantic Knowledge Representation and Reasoning

Driving in complex urban environments requires reasoning about the semantic meaning of road elements and their relations, not only about geometry. Early cognitive-robotics work introduced spatial hierarchies that combine metric and symbolic abstraction [17], ontology frameworks modeled object–place relations and task context for service robots [18], and knowledge-sharing systems explored reuse across robots via cloud databases [19]. These ideas consolidated into standardized semantic technologies in which classes and properties are written in OWL [20], rules in SWRL [21], and inference is carried out by description-logic reasoners such as Pellet [22] or programmatic engines such as Owlready2 [23], with explainability as the central advantage because conclusions can be traced to the premises that entailed them [24]. TOSM organizes environmental knowledge for context-aware navigation and multi-robot task allocation [4,5], and has since been applied on-road to object-centric pose estimation [6] and to context-aware localization with adaptive sensor fusion [7]. Across this line, however, the semantic layer served perception consistency and high-level task allocation and stopped at the symbol level: it was never used to select on-road driving behavior or to emit an executable trajectory, so the meaning it captured could not reach the control layer. This work retains the TOSM foundation and its measurable attributes but extends the same representation upward into behavior reasoning and downward into trajectory generation, so that the inferred meaning reaches the vehicle’s motion.

### 2.3. Semantic Maps for Autonomous Driving

Map representations for mobile robots evolved from occupancy grids [25] and feature-based maps [26] to dense point-cloud and topological forms [27]. For structured on-road driving, HD maps provide lane-level geometry and topology in standardized formats such as Lanelet2 [28] and OpenDRIVE [29], with broad overviews of their construction [30,31]. Because manual maps are costly to maintain, online methods learn vectorized map elements directly from on-board sensors [32,33,34]. To add meaning, semantic mapping and semantic SLAM attach class labels and object structure to geometric maps [35], through dense per-pixel fusion [36], LiDAR semantic registration [37], and segment-level descriptors for place recognition [38], and multi-hierarchical semantic maps couple spatial layers with symbolic reasoning [39,40]. These maps are accurate and increasingly semantic, yet their semantics are attached as passive labels rather than organized into a reasoning-ready relational model that exposes why an element matters and which maneuver it implies, so the map cannot itself drive a decision. This work converts a standardized HD map [28] into a TOSM-based semantic HD map whose elements carry symbolic, explicit, and implicit attributes and explicit relations, turning the map from a geometric backdrop into the substrate on which behavior is inferred.

### 2.4. Behavior Reasoning and Symbolic Mission Planning

Once a situation is interpreted, the system must both decide the discrete driving behavior (yielding, stopping, following, changing lanes) and sequence such behaviors toward a goal. Ontology-based methods model the urban scene in OWL and use a logic reasoner to generate legally and safely valid maneuvers [41], reason over spatio-temporal context for driving assistance [42], combine ontology reasoning with deep perception so that traffic rules compensate for the opacity of black-box predictors [43], and, more recently, use large language and vision-language models as a flexible source of world knowledge for scene reasoning and decision-making [44,45] or couple knowledge-graph reasoning with language models for explainable behavior prediction [46]. A parallel line specifies driving behavior as prioritized or formally verifiable traffic rules: rulebooks that pre-order competing objectives [47] and traffic rules formalized in higher-order logic for accountability [48]. This line, however, stops at specifying or checking behavior rather than turning it into a trajectory. Sequencing is addressed by symbolic planning with precondition–effect action models, from STRIPS [49] to PDDL [50], solved efficiently by heuristic-search planners such as Fast Downward [51,52] and applied to automated driving to order maneuvers between routing and tracking [53]. Hybrid extensions add numeric processes and events for continuous dynamics [54], middleware such as ROSPlan embeds planning in closed-loop execution [55], and hierarchical multi-robot task planning has been posed over TOSM [5]. Two limitations run through this body of work: the inferred decision is rarely connected to *how* it becomes a kinematically feasible trajectory, and the planning problem is typically constructed by hand or extracted from perception in an ad hoc way, leaving a gap between the situation model and the planner’s input. This work derives behaviors from SWRL rules over the TOSM map and generates the PDDL mission problem directly from the inferred semantic state, so that inferred hazards gate the relevant lanes and force handling behaviors into the plan by necessity rather than by manually specified goals, and the resulting plan and behaviors are then handed to the trajectory optimizer within the same representation.

### 2.5. Local Motion Planning and Trajectory Optimization

Local motion planning turns the chosen maneuver and drivable corridor into a smooth, kinematically feasible trajectory. Sampling-based planners grow a tree of feasible motions [10] and can be made asymptotically optimal [11] but yield non-smooth paths with no natural way to trade competing soft objectives, and search-based planners over motion primitives guarantee resolution-completeness [12] at a cost that grows sharply with resolution. Optimization-based planners instead represent the trajectory with few parameters and minimize a cost balancing smoothness, feasibility, and task objectives [56,57], which suits on-road driving where lane geometry already constrains the corridor. Within this family, Gaussian Process Motion Planning represents the continuous trajectory as a sample from a Gaussian process and expresses every requirement as a factor on a factor graph, recovering the trajectory as the maximum-a-posteriori solution of one nonlinear least-squares problem [58,59]. The Gauss–Markov prior yields an exactly sparse system re-solved incrementally with incremental smoothing and mapping [60,61] in the GTSAM library [62], and the same machinery supports joint planning, control, and estimation in dynamic settings [63,64], real-time semantic and Gaussian-process planning in traffic [65,66], and even high-speed racing [67]. This factor-graph formulation also underlies our group’s localization layer [7], which motivates reusing the same inference machinery. A body of work does couple behavioral decisions to trajectory generation: semantic corridors encode discrete decisions such as yielding, stop lines, and lane changes as spatio-temporal constraints for an optimizer [68], interaction-aware systems such as EPSILON select a policy and hand it to an optimization layer [69], game-theoretic planners blend data-driven behavior priors with optimization [70], and multipolicy decision-making evaluates closed-loop policy rollouts [71]. These couplings, however, are pairwise and bespoke: a decision module feeds a planner through a predefined interface, and the decision itself is not drawn from an auditable ontological model shared with the map and the localization layer. Absent such a layer, factor-graph optimizers typically consume undifferentiated geometric inputs rather than the output of an explicit reasoning layer, so a stop line, a yielding merge, and a static curb are all handled as anonymous geometry. This work attaches a semantic lane-centerline factor and lightweight interaction factors to the factor graph and raises the routing cost on the semantic map, so that the behavior inferred upstream is realized as a graded, kinematically feasible response of lateral avoidance, longitudinal deceleration, and lane-change detour, produced by the same incremental factor-graph machinery used for localization.

## 3. Semantic Reasoning and Mission Planning

**Figure 1:**
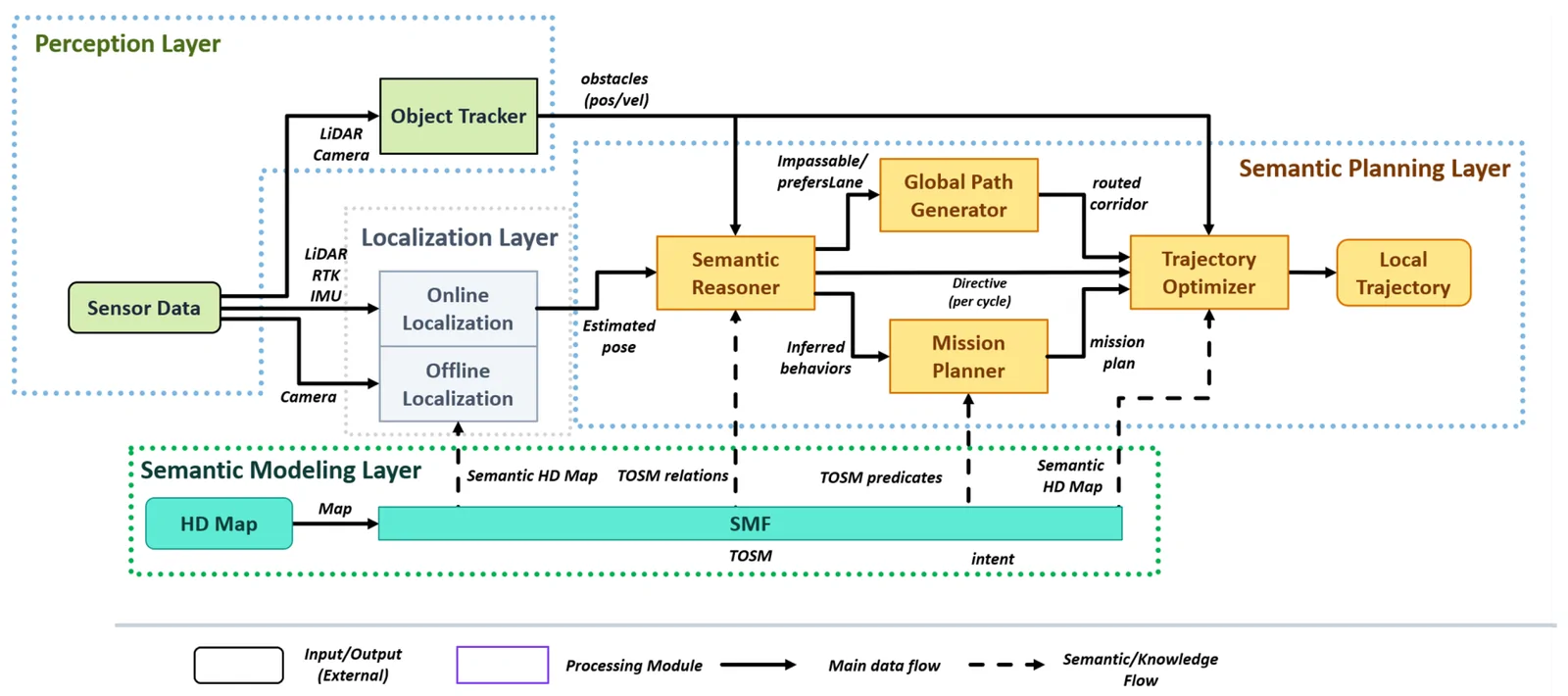
System overview.

Before detailing the semantic layers, Figure 1 places them in the full framework. The system comprises four layers (perception, localization, semantic modeling, and semantic planning) connected by a main data flow (solid) that carries metric quantities and a semantic/knowledge flow (dashed) that shares one semantic model across the stack. The contributions of this paper are the last two: the *semantic modeling layer*, which converts a standardized HD map into the TOSM-based semantic HD map through the semantic modeling framework (SMF), and the *semantic planning layer*, whose semantic reasoner, mission planner, and factor-graph trajectory optimizer turn that map into behavior, a mission route, and a local path. The perception layer and the localization layer (supplying the ego pose, from our prior work [7]) are used as-is and enter only as inputs to the planning layer. The framework assumes a standardized HD map as input. Map construction, online update, and map-degraded operation fall within the scope of the mapping subsystem. Crucially, the semantic HD map is not a one-shot input but is broadcast as a knowledge flow to the reasoner, the mission planner, and the trajectory optimizer alike, so the meaning it carries reaches every stage from decision to motion. This section develops the semantic modeling and reasoning parts. The factor-graph trajectory optimizer follows in Section 4.

The layer developed in this section turns the perceived world, expressed against the TOSM-based semantic HD map, into two products that the local trajectory optimizer of Section 4 consumes: a set of *behavioral directives* (stop, yield, lane change, reroute) attached to map places, and a *mission route*, a connected sequence of lanes from the ego to its destination that respects the inferred constraints. The design goal is a clean separation of concerns: perception supplies boolean qualitative predicates, a compact symbolic rule base draws behavioral conclusions from them, and a symbolic planner turns the conclusions into a mission. No numeric threshold appears inside a reasoning rule, a separation detailed in Section 3.2.

### 3.1. TOSM-Based Semantic Map

**Figure 2:**
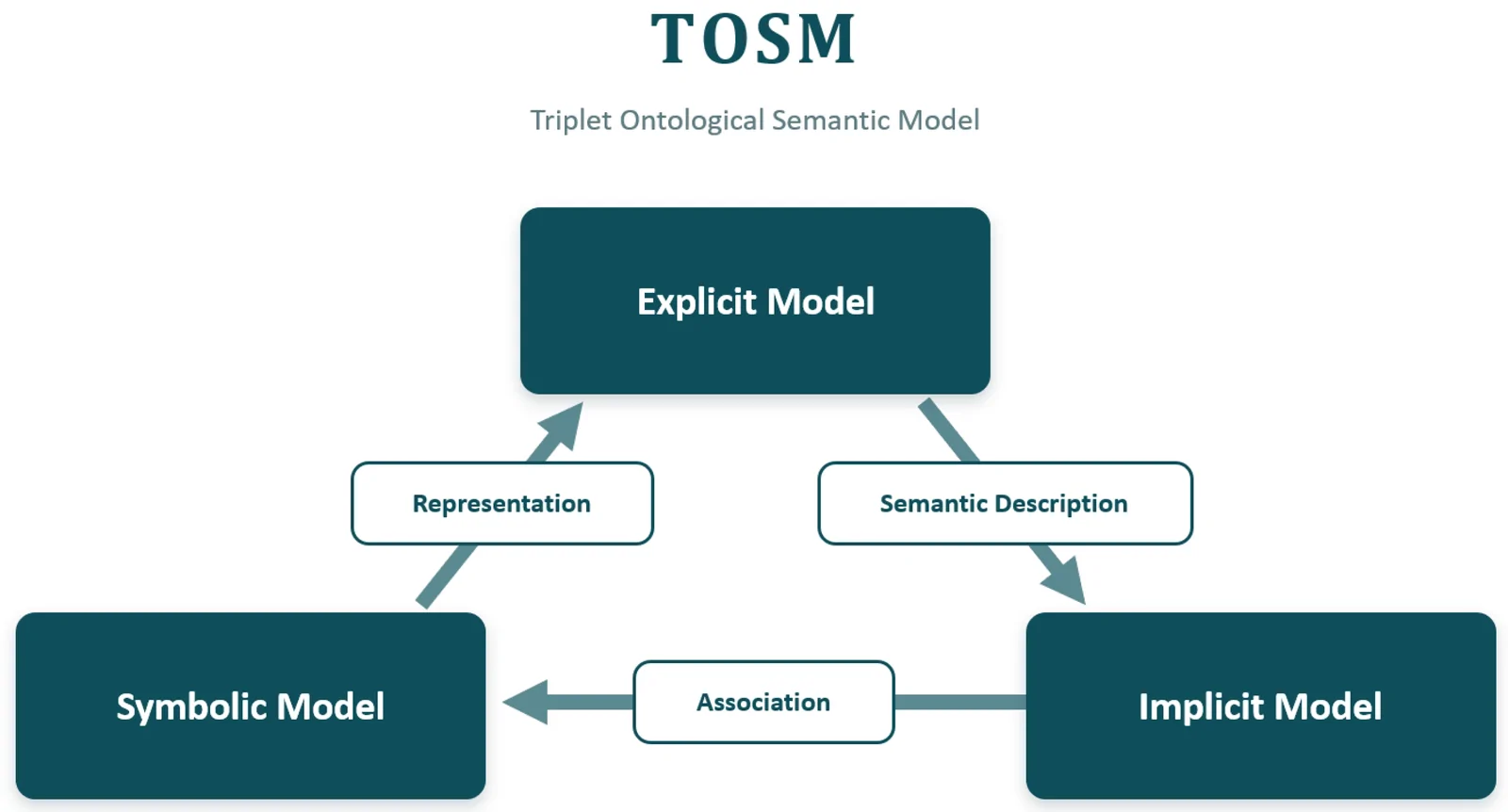
The Triplet Ontological Semantic Model (TOSM).

TOSM [4] expresses each environmental element through three coupled models (Figure 2): a *symbolic* model carrying its ontological identity and the relations a reasoner manipulates, an *explicit* model holding its metric geometry, and an *implicit* model of attributes derived or inferred from context, tied together by *representation*, *semantic description*, and *association*.

The map is an instance of the TOSM ontology, whose top concept *EnvironmentalElement* specializes into four branches, *Object*, *Place*, *Robot*, and *Sensor*. Table 2 lists the class hierarchy used by the reasoner. Perceptible entities become *Object* individuals (a crossing pedestrian is a *Person*, a merging car a *Vehicle*, a signal a *TrafficLight*, and a detection with no finer label an *UnknownObject*). The road network becomes *Place* individuals (each drivable link a *RoadSegment*, each pedestrian region a *Crosswalk* or *Sidewalk*). The ego vehicle forms the *Robot* branch, and its sensors a separate *Sensor* branch linked to the robot by isEquipped rather than modeled as a kind of robot.

*Object properties* relate individuals and carry the spatial, topological, and mission structure the reasoner reads, together with the relations it infers. Table 3 lists them with their domain, range, and logical characteristics as declared in the ontology.

The scale of the resulting Busan map and its logical consistency are reported with the experimental setup in Section 5.1.3. The offline conversion assigns each link a speedLimit from its road type, which the planner reads directly as its per-cycle speed bound (Section 3.3). An OWL-DL reasoner [22] confirms the converted map contains no unsatisfiable class before any behavioral reasoning is applied.

### 3.2. Rule-Based Behavioral Inference

Behavior is inferred by a compact rule base of nine rules, listed below. The rules are DL (Description Logic)-safe and are evaluated at run time by forward chaining over the materialized facts under a closed-world, unique-name reading. On their positive fragment, this coincides with SWRL entailment, while the derived isPassable=¬isBlocked below uses negation-as-failure, which SWRL itself does not provide but which is materialized as a positive assertion at the perception interface before either reasoner runs. The DL reasoner is never in the run-time loop. Offline it serves the map-consistency check and, on this positive fragment, cross-validates the run-time engine’s behavioral entailments (Section 5.2). The rules are grouped into behavioral rules that produce driving directives (B-1–B-6), an event rule that flags a mission-level reroute (E-1), and passability rules that supply the mission planner (P-1, P-2). Rules marked “(optional)” are enabled by a single switch, so the same base can be run in a conservative or a permissive configuration without editing individual rules. In every rule, the variables bind individuals of the ontology of Table 2: ?r denotes the ego (a *Robot*), ?o an environmental *Object* such as a pedestrian, a vehicle, or a traffic light, and ?l and ?p denote *Place*s (lanes or regions). A numeric subscript distinguishes co-occurring individuals of the same kind, so that $?l_{1}$ is the ego lane while $?l_{2}$ and $?l_{3}$ are adjacent lanes, and $?p_{1},?p_{2}$ are a place and its successor. This rule base is a core demonstration of the substrate and its grounding, not a complete urban-driving policy, and the contribution is this reasoning-to-motion architecture rather than the particular rule set. Because the rules are declarative over the materialized predicates, coverage can be scaled by declaring further rules over the same substrate, whether hand-authored or drawn from a formalized traffic code, to reach richer right-of-way negotiation, occlusion, and complex intersection topologies. Adding such rules leaves the reasoning engine, the grounding interface, and the trajectory layer unchanged. Formalizing a complete traffic-rule base is a separate knowledge-engineering effort, orthogonal to the integration studied here. As an interface check, a further right-of-way rule (B-7, yield to crossing traffic at an unsignalized intersection) was declared over the existing predicates: it required no change to the reasoning engine, the grounding interface, or the trajectory layer, inferred shouldYieldTo as intended, and left every existing scenario outcome unchanged. On the semantic boundary, the behavioral and event rules (B-1–B-6, E-1) use only positive atoms, so their run-time entailments coincide with the open-world SWRL entailments and are monotone under added facts. The sole negation-as-failure element, isPassable=¬isBlocked, is materialized as a positive assertion at the perception interface before reasoning. The unique-name assumption is sound because each map individual carries a distinct HD-map IRI. The relation isNextTo and the lateral relations isLeftOf/isRightOf are regulatory and topological assertions from the HD map rather than geometric proximity tests. Here, isNextTo records that a control device regulates a lane’s stop line, and lateral adjacency records a legal lane-change target (same travel direction, crossable boundary). Rule B-3 therefore fires only for the signal that governs the ego lane, and lane-change routing proposes only legal maneuvers.

- **Rule B-1 (Stop for a crossing pedestrian).** The ego stops for a pedestrian on its lane whose intention is crossing and whose approach is imminent.
  (1) $$\begin{array}{cc} \; & \mathrm{Person}(?o)\wedge \mathrm{Robot}(?r)\wedge \mathrm{isLocatedAt}(?r,?l)\wedge \mathrm{isInsideOf}(?o,?l)\\ \; & \wedge \;\mathrm{intention}(?o,\mathrm{crossing})\wedge \mathrm{isImminent}(?o)\;\to \;\mathrm{requiresStopFor}(?r,?o) \end{array}$$
- **Rule B-2 (Stop for a critical object).** The ego stops for any object on its lane that is flagged critical.
  (2) $$\begin{array}{cc} \; & \mathrm{Object}(?o)\wedge \mathrm{Robot}(?r)\wedge \mathrm{isLocatedAt}(?r,?l)\\ \; & \wedge \;\mathrm{isInsideOf}(?o,?l)\wedge \mathrm{isCritical}(?o)\;\to \;\mathrm{requiresStopFor}(?r,?o) \end{array}$$
- **Rule B-3 (Stop at a red traffic light).** The ego stops when its lane is next to a traffic light in the red state.
  (3) $$\begin{array}{cc} \; & \mathrm{Robot}(?r)\wedge \mathrm{isLocatedAt}(?r,?l)\wedge \mathrm{isNextTo}(?l,?o)\\ \; & \wedge \;\mathrm{TrafficLight}(?o)\wedge \mathrm{trafficLightState}(?o,\mathrm{red})\;\to \;\mathrm{requiresStopFor}(?r,?o) \end{array}$$
- **Rule B-4 (Prefer a clear adjacent lane).** When its own lane is congested and an adjacent lane is clear, the ego prefers that lane.
  (4) $$\begin{array}{cc} \; & \mathrm{Robot}\left(?r\right)\wedge \mathrm{isLocatedAt}\left(?r,?l_{1}\right)\wedge \mathrm{isCongested}\left(?l_{1}\right)\\ \; & \wedge \;\mathrm{isAdjacentLane}\left(?l_{2},?l_{1}\right)\wedge \mathrm{isClear}\left(?l_{2}\right)\;\to \;\mathrm{prefersLane}\left(?r,?l_{2}\right) \end{array}$$
- **Rule B-5 (Yield for a merging vehicle).** The ego yields to an approaching vehicle whose intention is merging.
  (5) $$\begin{array}{cc} \; & \mathrm{Robot}(?r)\wedge \mathrm{Vehicle}(?o)\wedge \mathrm{intention}(?o,\mathrm{merging})\wedge \mathrm{isApproaching}(?o)\\ \; & \;\to \;\mathrm{shouldYieldTo}(?r,?o) \end{array}$$
- **Rule B-6 (Prefer the lane away from the merge side, optional).** When a vehicle merges from one adjacent lane, the ego prefers the other, clear adjacent lane.
  (6) $$\begin{array}{cc} \; & \mathrm{Robot}(?r)\wedge \mathrm{Vehicle}(?o)\wedge \mathrm{intention}(?o,\mathrm{merging})\wedge \mathrm{isApproaching}(?o)\\ \; & \wedge \;\mathrm{isInsideOf}\left(?o,?l_{3}\right)\wedge \mathrm{isLocatedAt}\left(?r,?l_{1}\right)\wedge \mathrm{isAdjacentLane}\left(?l_{3},?l_{1}\right)\\ \; & \wedge \;\mathrm{isAdjacentLane}\left(?l_{2},?l_{1}\right)\wedge \left(?l_{2}\neq ?l_{3}\right)\wedge \mathrm{isClear}\left(?l_{2}\right)\;\to \;\mathrm{prefersLane}\left(?r,?l_{2}\right) \end{array}$$
- **Rule E-1 (Reroute on a blocked route place, optional).** A reroute is flagged when a place on the current route becomes impassable.
  (7) Robot(?r)∧isOnRoute(?r,?p)∧isBlocked(?p)→needsReroute(?r)
- **Rule P-1 (Move to a connected passable place).** A place connected to the ego place and passable is admissible for the mission planner.
  (8) $$\begin{array}{cc} \; & \mathrm{Robot}\left(?r\right)\wedge \mathrm{isLocatedAt}\left(?r,?p_{1}\right)\wedge \mathrm{isConnectedTo}\left(?p_{1},?p_{2}\right)\wedge \mathrm{isPassable}\left(?p_{2}\right)\\ \; & \;\to \;\mathrm{canMoveTo}(?r,?p_{2}) \end{array}$$
- **Rule P-2 (Avoid a blocked place, optional).** A blocked place is excluded from the mission.
  (9) Robot(?r)∧Place(?p)∧isBlocked(?p)→mustAvoid(?r,?p)

The central design decision is that a rule never compares a number. Every predicate a rule reads (isImminent, isCritical, isApproaching, isCongested, isClear, isBlocked, isPassable) is a boolean produced by a separate perception interface that owns all thresholds. The time-to-collision bands are nested by construction, isCritical(TTC<1s)⊂isImminent(TTC<3s)⊂isApproaching(TTC<6s), so, on the time-to-collision axis, the stop-triggering band is nested inside the yield-triggering one. Congestion is banded from a discrete level (isClear<2≤isCongested, with isHeavilyCongested at the top level, ≥3). Finally, passability is derived, isBlocked=isRestricted∨hasSevereEvent∨isHeavilyCongested with isPassable=¬isBlocked (a closed-world negation). Moving the thresholds out of the rules makes the symbolic layer *syntactically* threshold-free and keeps it auditable: a fired rule is explained by which boolean predicates held, and because every threshold lives at this single interface rather than being scattered across the rule base, re-tuning one moves only where a band boundary falls, never the rule structure. The conclusions of course remain functions of these constants. The interface localizes the constants but does not remove them. To reject single-frame false positives, a predicate is materialized as true only after it persists for *K* consecutive perception frames, a debounce guard at this same interface. Because a directive grounds into the speed-cap factor as a time-extended deceleration rather than a hard stop, a transient misfire yields bounded, recoverable braking rather than phantom emergency braking. Full probabilistic reasoning inside the symbolic layer, for example confidence-weighted predicates propagated to a soft directive stiffness, is a natural extension and is left as future work.

### 3.3. Grounding Inferences into the Planner

Each inferred relation is grounded into an input that a specific numeric factor of Section 4 consumes, as listed in Table 4. A requiresStopFor conclusion becomes a stop directive placed at the object (for a pedestrian, the stop point is pulled back by a fixed safety margin), which sets the stop bound of the speed-cap factor. A shouldYieldTo conclusion becomes a yield directive carrying a speed cap taken from the yielded-to vehicle, which sets the yield bound of the same factor. The prefersLane conclusion is a lane preference folded into the mission-route cost (Section 4.6), and the resulting centerline is what the lane factor F7 (Section 4) then tracks. The unary boolean flag needsReroute (a data property rather than a binary relation) triggers a mission replan at the run-time coupling of Section 3.5. The two passability relations (canMoveTo, mustAvoid) are not trajectory directives but constraints for the mission planner of Section 3.4. The speed limit is deliberately not a rule conclusion: it is a static map attribute read directly from the ego link, so a routine bound never occupies the rule base.

### 3.4. Mission Planning as Symbolic Search

The mission (driving from the ego place to the destination while respecting the inferred passability and hazard constraints) is expressed as a classical planning problem in a compact PDDL domain and solved by symbolic search. The domain types (Listing 1) mirror the ontology of Table 2, and its predicates (Listing 2) are the machine-readable images of the map relations and of the behavioral conclusions the mission layer consumes (requiresStopFor, shouldYieldTo, canMoveTo, mustAvoid). The two remaining conclusions, prefersLane and needsReroute, are realized by the trajectory and routing layers (Table 4) and so have no predicate here. Its actions (drive, arrive, stop_for, and yield_to) and their essential pre/postconditions are given in Listing 3. The only goal is (reached_goal ego). Hazard handling is not written into the goal but forced by the preconditions: the drive action from a place is admissible only if every hazard at that place has been handled by a preceding stop_for or yield_to, and only along isConnectedTo successions that are canMoveTo and not mustAvoid. A valid plan is therefore a hazard-gated drive sequence, and reading off its drive steps yields the mission route as a connected lane sequence.

**Listing 1.** PDDL domain types, based on the TOSM class hierarchy of Table 2.
(:types

 object place robot sensor

- environmental_element

 road_user landmark unknown_object

- object

 person vehicle bicycle

- road_user

 traffic_light sign surface_mark facility

- landmark

 hd_place semantic_place pedestrian_place

- place

 road_segment intersection

- hd_place

 unit_place

- semantic_place

 sidewalk crosswalk

- pedestrian_place

 ego

- robot

 camera lidar radar imu gnss

- sensor)


**Listing 2.** PDDL predicates: map relations, ego-state markers, and the images of the SWRL
conclusions.
;; spatial / connectivity

(is_connected_to

?from - place ?to - place)

;; ego state and progress markers

(is_located_at

?e - ego ?p - place)

(has_destination

?e - ego ?p - place)

(handled

?e - ego ?o - object)

(has_stopped_for

?e - ego ?o - object)

(has_yielded_to

?e - ego ?o - road_user)

(reached_goal

?e - ego)

;; hazard placement (asserted at problem generation)

(hazard_at

?o - object ?p - place)

;; inferred behavior consumed by the mission layer (images of SWRL conclusions)

(requires_stop_for

?e - ego ?o - object)

(should_yield_to

?e - ego ?o - road_user)

(can_move_to

?e - ego ?p - place)

(must_avoid

?e - ego ?p - place)


**Listing 3.** Actions of the mission-planning domain. Hazard handling is enforced through the drive
precondition rather than stated as a goal.
(:action drive

 :parameters (?e - ego ?from - place ?to - place)

 :precondition (and (is_located_at ?e ?from) (is_connected_to ?from ?to)

            (can_move_to ?e ?to) (not (must_avoid ?e ?to))

            (forall (?o - object)

              (imply (hazard_at ?o ?from) (handled ?e ?o))))

 :effect (and (not (is_located_at ?e ?from)) (is_located_at ?e ?to)))

(:action arrive

 :parameters (?e - ego ?p - place)

 :precondition (and (is_located_at ?e ?p) (has_destination ?e ?p)

            (forall (?o - object)

              (imply (hazard_at ?o ?p) (handled ?e ?o))))

 :effect (reached_goal ?e))

(:action stop_for

 :parameters (?e - ego ?o - object ?p - place)

 :precondition (and (is_located_at ?e ?p) (requires_stop_for ?e ?o)

            (hazard_at ?o ?p) (not (handled ?e ?o)))

 :effect (and (has_stopped_for ?e ?o) (handled ?e ?o)))

(:action yield_to

 :parameters (?e - ego ?o - road_user ?p - place)

 :precondition (and (is_located_at ?e ?p) (should_yield_to ?e ?o)

            (hazard_at ?o ?p) (not (handled ?e ?o)))

 :effect (and (has_yielded_to ?e ?o) (handled ?e ?o)))


    The problem is emitted over a bounded corridor around the ego–destination pair: the connectivity, passability, and canMoveTo facts are materialized over that corridor (not only for the ego’s immediate successors, as a single forward-chaining pass would produce), so that multi-step drive plans are well-formed, and the result is handed to a symbolic planner. Any sound classical planner suffices. The implementation selects, in order of availability, the heuristic-search planners of Helmert [51] and of Hoffmann and Nebel [72], falling back to a self-contained goal-count best-first search when neither is installed, so the mission layer carries no hard dependency on an external solver. If symbolic search fails for a query, a breadth-first route over the connectivity graph that skips mustAvoid places and forces the required waypoints provides a guaranteed fallback.

### 3.5. Run-Time Metric–Symbolic Coupling

At run time a coupling process closes the loop between the metric world and the symbolic layer. Each cycle, it (i) matches the ego UTM pose to a map link, (ii) assembles a scenario description: ego location, surrounding objects with their qualitative predicates, traffic-light states, and per-place attributes, (iii) fires the rule base and extracts the inferred relations, (iv) grounds them into the directives of Table 4, and (v) returns, together with the directives, the mission route and its resampled centerline waypoints. To bound cost, the mission plan is recomputed only on a semantic event (a destination change, a change in the mustAvoid set, a needsReroute conclusion, or the ego leaving its route) rather than on every cycle, whereas the directives are refreshed every cycle so that a newly perceived hazard enters the trajectory optimizer without delay. The resulting directives and route are exactly the inputs assumed by the factor graph of Section 4.

## 4. Factor-Graph-Based Local Trajectory Optimization

The semantic reasoning and planning of the preceding section infers what the ego vehicle should do, but not an executable motion. This section introduces a local trajectory planner that turns those decisions and the semantic HD-map structure into a smooth, kinematically feasible trajectory by formulating planning as inference over a factor graph, deliberately sharing the machinery of our group’s localization work [7]: whereas that work estimated where the vehicle is, the same machinery here determines how it should move within the drivable corridor. Of the sampling-, search-, and optimization-based families reviewed in Section 2.5, the optimization-based paradigm best suits structured on-road driving, where the corridor is already fixed by lane geometry and the task is to shape a smooth trajectory that respects kinematics and reacts to traffic.

The planner is built on the optimization-based, probabilistic-inference formulation of Gaussian Process Motion Planning [58,59], whose derivations and guarantees are established in that line of work and are adopted here without modification. The trajectory is a sparse set of support states linked by a Gaussian process prior, all objectives are factors on those states, and the trajectory is obtained by solving a single nonlinear least-squares problem with the incremental smoothing method of Kaess et al. [60], expressed on the factor-graph formalism of Dellaert [62]. Three properties make this formulation attractive. It is modular, because each requirement is an independent residual that can be added or reweighted without altering the solve. It is probabilistically grounded for its Gaussian factors, whose squared residual is a negative log-likelihood with confidence expressed as a covariance rather than an ad hoc gain. The one-sided hinge factors introduced below are not normalizable likelihoods but exterior penalties written in the same whitened least-squares form, where the covariance plays the role of an interpretable relative weight. It is efficient, because the Gauss–Markov structure of the prior yields an exactly sparse system that is re-solved incrementally. This sparsity and its incremental re-solution are proven in the cited works, so those guarantees carry over unchanged to the present planner. The inference method itself is not a contribution of this work [66,67]. The contribution is the integration. The same set of factors is driven by the semantic HD map and the symbolic mission plan, is extended with the dynamic interaction factors introduced below, and shares the incremental inference machinery of the localization layer [7]. Each factor below is attributed to its origin: the terms taken from the established formulation are used exactly as proven there, and the terms original to this work are derived in full, with closed-form residual Jacobians supplied to the solver.

### 4.1. Trajectory Representation and Optimization

This subsection fixes the representation on which every factor below acts: the continuous-time state and its manifold retraction, the Gaussian-process prior that couples consecutive states, the interpolation that densifies the output, and the maximum-a-posteriori inference that solves for the trajectory.

#### 4.1.1. Continuous-Time State Representation

N+1 support states, sampled at a fixed time interval Δt over a planning horizon T=NΔt, parameterize the local trajectory. The state at the *k*-th support node is defined as

(10) $$\xi_{k}=\left(\;x_{k},\;v_{k}\;\right),\;k=0,1,\ldots ,N,$$

where $x_{k}=\left(x_{k},y_{k},\theta_{k}\right)\in SE\left(2\right)$ is the planar pose and $v_{k}=\left({\dot{x}}_{k},{\dot{y}}_{k},{\dot{\theta }}_{k}\right)$ the velocity, expressed as world-frame components in the planning frame introduced below. Modeling velocity as an explicit state, rather than finite-differencing poses, is essential because the GP prior of Section 4.1.2 and the longitudinal factors of Section 4.2 and Section 4.4 reference it directly.

The pose component is not a vector: SE(2) is a Lie group, so each state lives on the smooth manifold $M=SE\left(2\right)\times R^{3}$ of dimension six and the full trajectory on the product $M^{N+1}$. A correction cannot be added to $\xi_{k}$ componentwise without violating the heading’s group structure. Optimization instead operates in the tangent space at the current estimate through the standard retraction $\xi_{k}⊕\delta_{k}=\left(x_{k}\cdot \mathrm{Exp}\left(\delta_{k}^{x}\right),\;v_{k}+\delta_{k}^{v}\right)$ with increment $\delta_{k}=\left(\delta_{k}^{x},\delta_{k}^{v}\right)\in R^{6}$ [61], where Exp:se(2)→SE(2) is the exponential map and its inverse Log recovers the minimal error between two poses [61,62]. All residuals and Jacobians below are therefore expressed in this six-dimensional tangent space, which is why the pose Jacobians of the custom factors are written in the Pose2 body frame rather than in raw (x,y,θ) coordinates.

The optimizer works in a vehicle-local frame with origin at the current pose and *x*-axis along the heading, so the ego pose maps to (x,y,θ)=(0, 0, 0). This is not mere convenience. In the global UTM frame, the coordinates reach $10^{5}$–$10^{6}$ m, and the resulting precision loss degrades the conditioning of each Gauss–Newton solve, occasionally rendering it indeterminate. Re-centering keeps all coordinates within tens of meters and restores conditioning. The goal and centerline hint are transformed in before optimization and the result back before the controller, while the velocity is stored as world-frame components $\left({\dot{x}}_{k},{\dot{y}}_{k}\right)$. Because the heading varies along the horizon, the forward speed used by the kinematic and speed factors below is the magnitude

(11) $$s_{k}\;\equiv \;∥v_{k}∥\;=\;\sqrt{{\dot{x}}_{k}^{\;2}+{\dot{y}}_{k}^{\;2}},$$

which the nonholonomic factor of Section 4.2 aligns with the heading by driving the body-lateral component $-{\dot{x}}_{k}\sin\theta_{k}+{\dot{y}}_{k}\cos\theta_{k}$ to zero. Using $s_{k}$ rather than ${\dot{x}}_{k}$ makes the forward speed exact at every state regardless of the local heading, and $s_{k}$ is the quantity bounded wherever a longitudinal speed appears below.

#### 4.1.2. Gaussian Process Trajectory Prior

In the continuous-time Gaussian-process formulation of trajectory optimization [58,59], the trajectory is a sample from a vector-valued Gaussian process $\theta \left(t\right)∼\mathrm{GP}(\mu \left(t\right),K\left(t,t^{\prime }\right))$, whose negative log $\frac{1}{2}{∥\theta -\mu ∥}_{K}^{2}$ is the smoothness cost. Generating the GP by a linear time-varying SDE with white-noise-on-acceleration [59,73] makes the precision block tridiagonal, so the prior cost decomposes into between-state factors

(12) $$f_{\mathrm{gp}}\left(\theta_{i},\theta_{i+1}\right)=\frac{1}{2}\;{∥\;\Phi \left(t_{i+1},t_{i}\right)\;\theta_{i}-\theta_{i+1}+u_{i,i+1}\;∥}_{Q_{i,i+1}}^{2},$$

with state-transition matrix Φ, integrated control $u_{i,i+1}$, and accumulated covariance $Q_{i,i+1}$. For the constant-velocity model $\theta_{k}=\left(x_{k},v_{k}\right)$, $\Phi \left(\Delta t\right)=\left[\begin{array}{cc} I & \Delta t\;I\\ 0 & I \end{array}\right]$ and $Q_{i,i+1}$ (position block $\frac{1}{3}\Delta t^{3}\;Q_{c}$) take the standard double-integrator closed forms, with $Q_{c}$ the acceleration-noise PSD (small enforces smoothness, large relaxes it; longitudinal and lateral tuned separately). These closed forms and the exactly-sparse block-tridiagonal precision are the sparse-GP result of [59,73] and are used here unchanged (exact in the vector-space parameterization, first-order on the SE(2) pose component). Since $Q_{i,i+1}$ shrinks as $\Delta t^{3}$, the support count is chosen jointly with the horizon, with finer output resolution from the interpolation of Section 4.1.3 [67].

#### 4.1.3. Gaussian Process Interpolation

A key property of the Gauss–Markov prior is that the trajectory, though parameterized by sparse support states, can be queried at any $\tau \in (t_{i},t_{i+1})$ in closed form as a linear combination $\theta \left(\tau \right)=\mu \left(\tau \right)+\Lambda \left(\tau \right)\left(\theta_{i}-\mu_{i}\right)+\Psi \left(\tau \right)\left(\theta_{i+1}-\mu_{i+1}\right)$ of the two adjacent states, with coefficients Λ(τ),Ψ(τ) from Φ and Q [59,73]. This interpolation is exact in the vector-space (tangent) parameterization, the pose component subject to the same first-order manifold linearization. This O(1) operation decouples optimization resolution from output resolution, so a coarse support spacing suffices: the controller receives a dense trajectory though the optimization spans few states. Whereas the general formulation also uses it for *interpolated obstacle factors* that densify the in-optimization collision check, the present planner attaches all factors to the support states and interpolates only the output, suited to on-road driving where the support spacing is already fine relative to the surrounding-vehicle geometry.

#### 4.1.4. Maximum a Posteriori Trajectory Inference

A factor graph is a bipartite graph over the variable nodes $\Theta =\{\xi_{0},\ldots ,\xi_{N}\}$ and the factor nodes F [61,62], with joint posterior $p\left(\Theta \right)\propto \prod_{c\in F}\phi_{c}\left(\Theta_{c}\right)$. Under a Gaussian model $\phi_{c}\propto \exp\left(-\frac{1}{2}{∥r_{c}∥}_{\sum_{c}}^{2}\right)$ with residual $r_{c}\left(\Theta_{c}\right)=h_{c}\left(\Theta_{c}\right)-z_{c}$, each factor’s negative log-likelihood is a whitened squared error. Casting motion planning as maximum-a-posteriori inference combines the prior of Section 4.1.2 with the feasibility likelihoods; under Gaussian factors this is exactly the nonlinear least-squares [58,59,61]

(13) $$\theta^{*}=\arg\max_{\theta }\;p\left(\theta \right)=\arg\min_{\theta }\sum_{c\in F}\frac{1}{2}\;{∥\;r_{c}\left(\theta \right)\;∥}_{\sum_{c}}^{2},\;{∥r_{c}∥}_{\sum_{c}}^{2}=r_{c}^{\;⊤}\sum_{c}^{-1}r_{c},$$

Every factor below is written in this form $f_{c}={∥r_{c}∥}_{\sum_{c}}^{2}$, so specifying the planner reduces to specifying each residual $r_{c}$ and its stiffness $\sum_{c}$; the factor classes are defined below and summarized in Table 5.

The objective (13) is solved by the Gauss–Newton iteration standard to factor-graph inference [61,62]: each residual is linearized in the tangent space with a closed-form Jacobian $H_{c}$, the increment solves the normal equations $\Omega \;\delta^{*}=-H^{\;⊤}\sum^{-1}r$, and the estimate is retracted until convergence. The information matrix $\Omega =\sum_{c}H_{c}^{\;⊤}\sum_{c}^{-1}H_{c}$ has the factor-graph adjacency as its sparsity, so with only the binary GP prior and ternary jerk terms coupling states it is banded (tridiagonal, pentadiagonal where jerk is active) and solved by sparse Cholesky in O(N) rather than $O(N^{3})$, as analyzed in Section 4.7.2.

Rather than re-solving from scratch each cycle, the problem is solved with incremental smoothing and mapping [60], which maintains the factorization as a Bayes tree and, on each update, relinearizes and re-solves only the affected variables. Because each cycle is re-expressed in a fresh vehicle-local frame (Section 4.7.2), this incrementality is exploited within a cycle (across relinearization iterations and by warm-starting from the previous trajectory) rather than by persisting the Bayes tree across cycles. The previous cycle’s trajectory initializes the current one $\theta^{\left(0\right)}$, and when the upstream global plan supplies it the routed centerline serves as the hint, so the local trajectory inherits the lane-level routing. Because several factors below are hinge-shaped and nonlinear, the initialization is preconditioned to respect the acceleration limits of Section 4.2, preventing a large initial residual that would cause the solver to reject the update.

**Figure 3:**
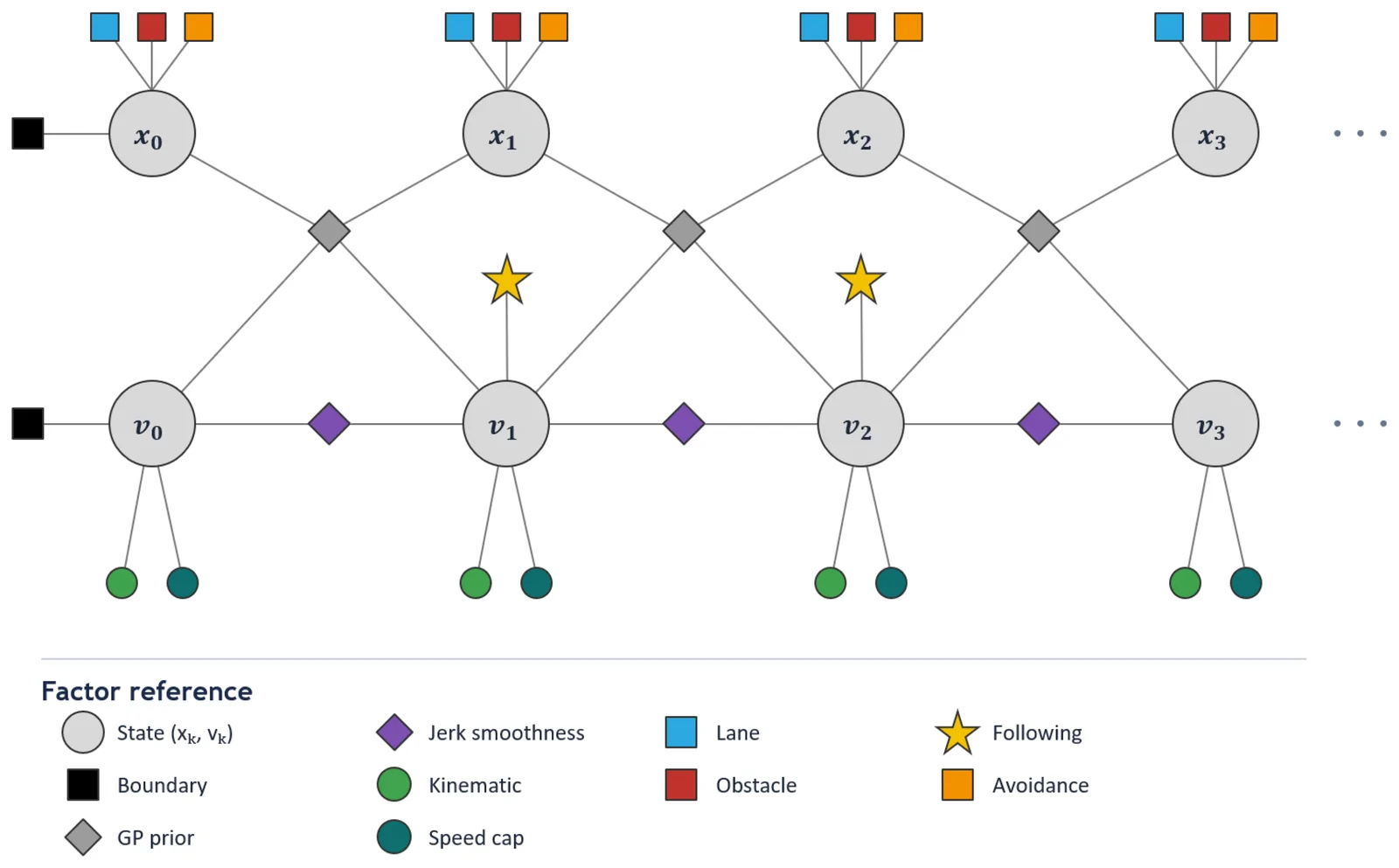
Factor-graph structure of the local trajectory optimizer.

Figure 3 shows the resulting graph. Each support state comprises a pose $x_{k}\in SE\left(2\right)$ (top row) and a velocity $v_{k}$ (bottom row), drawn as gray circles, with boundary factors pinning the initial state. Each pose carries the unary lane-centerline, obstacle, and lateral-avoidance factors; each velocity carries the unary kinematic and speed-cap factors. The GP prior (gray) is binary on consecutive states, the jerk-smoothness factors (purple) act along consecutive velocities, and the longitudinal-following factor (star) couples a velocity to its pose. The GP prior’s Gauss–Markov structure makes the graph sparse and banded, enabling efficient incremental inference.

### 4.2. Vehicle Kinematic Factors

Kinematic factors on each support state keep the trajectory executable by a nonholonomic vehicle, as in the dynamics-consistency constraint of [7]. They are soft, not hard: hard constraints would need a constrained optimizer incompatible with the incremental least-squares solve, whereas soft factors degrade gracefully under momentary conflict, with each covariance set so the factor dominates only near infeasibility.

#### 4.2.1. Boundary Factors

Prior factors anchor the endpoints: the initial pose and velocity to the current vehicle state, the terminal pose and velocity to the look-ahead goal from the global plan:

(14) $$f_{\mathrm{start}}\left(\xi_{0}\right)={∥\xi_{0}-{\hat{\xi }}_{\mathrm{start}}∥}_{\sum_{0}}^{2},\;f_{\mathrm{goal}}\left(\xi_{N}\right)={∥\xi_{N}-{\hat{\xi }}_{\mathrm{goal}}∥}_{\sum_{N}}^{2}.$$

The start factor is tight, clamping the first state to the measured pose and velocity for continuity with current motion. The goal factor is looser, since the look-ahead goal is only a guidance point the surrounding-vehicle factors may displace.

#### 4.2.2. Nonholonomic Factor

A wheeled vehicle cannot translate laterally. Its body-frame lateral velocity must vanish:

(15) $$f_{\mathrm{nh}}\left(\xi_{k}\right)={∥\;v_{y,k}^{\;\mathrm{body}}\;∥}_{\sigma_{\mathrm{nh}}}^{2},\;v_{y,k}^{\;\mathrm{body}}=-{\dot{x}}_{k}\sin\theta_{k}+{\dot{y}}_{k}\cos\theta_{k},$$

where $v_{y,k}^{\;\mathrm{body}}$ is the body-frame lateral velocity, coupling heading and velocity direction to rule out sideways sliding. The stiffness $\sigma_{\mathrm{nh}}$ is tuned jointly with the GP prior, which already penalizes pose–velocity inconsistency.

#### 4.2.3. Lateral Acceleration

Lateral acceleration $a_{\mathrm{lat}}=s_{k}\;{\dot{\theta }}_{k}$ has no dedicated factor; it is shaped indirectly by the curvature-adaptive cap of the speed-cap factor (Section 4.5, Equation (22)), a soft cap biasing the speed downward in curves toward the comfort limit $a_{\mathrm{lat}}^{\max}\;\approx \;0.3$ g [74], whose limited authority on fast recorded passes is quantified in Section 5.8. A separate lateral-acceleration hinge on top over-constrained the yaw rate and conflicted with the boundary velocity, so the planner relies on the speed cap alone and a hard comfort bound is left as future work. When the forward speed is instead a free variable, as in the actuated closed-loop evaluation of Section 5.12, this soft cap binds and holds the lateral acceleration toward the comfort target. Its limited authority is therefore a property of the fixed-velocity boundary in open-loop replay, not of the soft formulation itself.

#### 4.2.4. Longitudinal Acceleration

Longitudinal acceleration is not given its own hinge factor. The white-noise-on-acceleration GP prior of Equation (12) already penalizes it through the coupling of consecutive velocities, so a separate factor is redundant and couples the same velocity pair, causing ill-conditioning without improving the trajectory. Smooth speed change is therefore left to the GP prior together with the jerk factors below. The comfort constant $a_{\mathrm{lon}}^{\max}\;\approx \;2\;m/s^{2}$ is retained only to pre-smooth the arc-length-sampled initialization, whose finite-difference velocities can jump between states, before optimization.

#### 4.2.5. Minimum-Turning-Radius Factor

**Figure 4:**
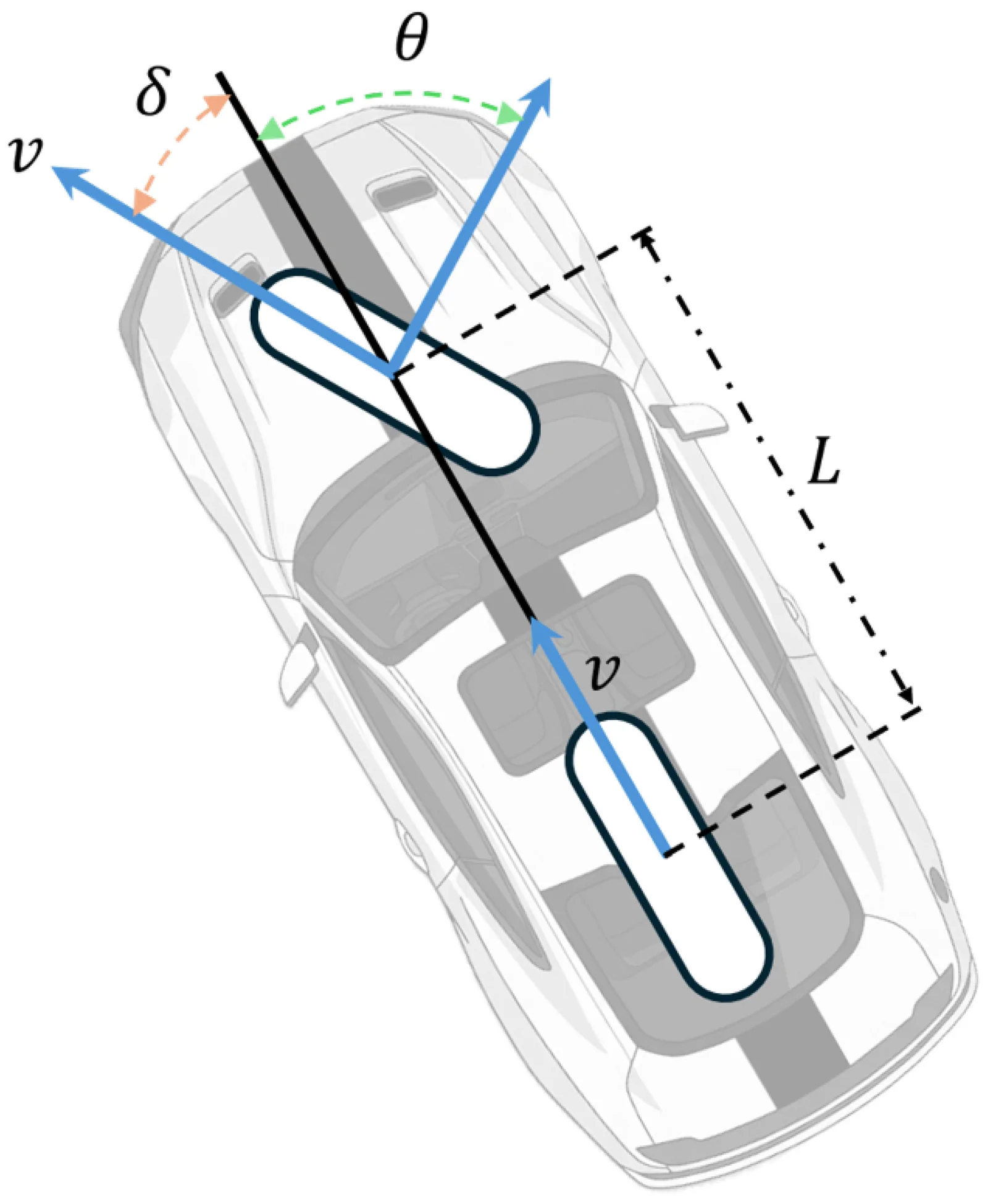
Kinematic bicycle model. *v* is the velocity at each axle, δ the front-wheel steering angle, θ the heading angle, and *L* the wheelbase.

From the kinematic bicycle model (Figure 4, wheelbase *L*) [75], the rear-axle geometry gives curvature κ=1/R=tanδ/L for steering angle δ, so $\delta \leq \delta_{\max}$ yields the minimum turning radius $r_{\min}=L/\tan\delta_{\max}$ and bounds the yaw rate $|{\dot{\theta }}_{k}|\;\leq \;s_{k}/r_{\min}$, imposed softly by

(16) $$f_{\mathrm{turn}}\left(\xi_{k}\right)={∥\;\max\;\left(0,\;|{\dot{\theta }}_{k}|-s_{k}/r_{\min}\right)\;∥}_{\sigma_{\mathrm{turn}}}^{2}.$$

It is hinge-shaped and silent in normal driving, active only in tight low-speed maneuvers [67]. Steering δ is not a state variable: the curvature $\kappa_{k}={\dot{\theta }}_{k}/s_{k}$ maps one-to-one to δ through κ=tanδ/L, so optimizing over $\left({\dot{\theta }}_{k},v_{k}\right)$ is equivalent, imposing only the curvature bound and the no-slip assumption (the latter from the nonholonomic factor of Equation (15)) and leaving the specific steering angle to the controller. The bicycle model thus supplies the kinematic constraints, not a forward integrator.

#### 4.2.6. Jerk-Smoothness Factors

Ride comfort is governed less by acceleration than by its rate of change. Two factors therefore penalize the jerk of each velocity channel as a second difference over three consecutive states,

(17) $$f_{\mathrm{jerk}}^{\left(c\right)}\left(v_{k-1},v_{k},v_{k+1}\right)={∥\;\frac{v_{k+1}^{\left(c\right)}-2\;v_{k}^{\left(c\right)}+v_{k-1}^{\left(c\right)}}{\Delta t^{2}}\;∥}_{\sigma_{\mathrm{jerk}}^{\left(c\right)}}^{2},$$

with c=lon the longitudinal-jerk factor (F11) acting on the forward speed and c=yaw the angular-jerk factor (F12) acting on the yaw rate. Being ternary on neighboring velocities, they preserve the banded structure of the graph. The same smoothness can equivalently be obtained by promoting the prior to a white-noise-on-jerk model [76], which augments each state with an acceleration channel; the explicit second-difference form is used by default because it adds the comfort term without enlarging the state. They are enabled by default but bind only when the jerk would otherwise be high, so on a trajectory the prior already keeps smooth, they contribute little and the ablation of Section 5.4 finds them dormant.

### 4.3. Lane Center-Line Factor

The kinematic factors ensure feasibility but do not keep the trajectory in-lane. The lane-centerline factor attracts each support state toward the centerline of the routed link. With $c(x_{k})$ the closest point on that centerline to $p(x_{k})$,

(18) $$f_{\mathrm{lane}}\left(\xi_{k}\right)={∥\;p\left(x_{k}\right)-c\left(x_{k}\right)\;∥}_{\sum_{\mathrm{lane}}}^{2}.$$

This mirrors the place-consistency factor of the prior localization work [7], tying the numerical trajectory to the road structure and transferring the upstream lane-level routing decision into the continuous trajectory. Attracting a trajectory toward a routed reference is standard on-road practice, from Frenet-frame generation [77] to the reference-line planners of [66,67]. Since the routed centerline already encodes any lane changes chosen by the global planner (Section 4.6), the attractor reproduces those maneuvers locally without an explicit lane-change primitive.

The closest point is the foot of the perpendicular from the support position onto the routed centerline polyline, so the residual reduces to the signed lateral offset from the lane [77]. Evaluating this per support state keeps the attractor valid through curves and across the junction between two routed links. When the global planner commits to a lane change, the routed centerline shifts over a few links. The per-state projection then yields a smoothly increasing offset. The optimizer balances this offset against the kinematic and interaction factors and tracks it as a feasible lane change rather than a jump.

One caveat arises when two adjacent lanelet centerlines are concatenated without lateral overlap: the sampled reference then contains a near-discontinuous step, which at a fine support-state spacing produces a large local residual that can destabilize the incremental solve. The reference is therefore preconditioned by an *angle-gated* smoother that relaxes only vertices whose turn angle exceeds a threshold, leaving nominal curvature untouched. This removes the step without rounding genuine curves, so lane keeping is unaffected while discontinuous lane-change references remain solvable. Its effect is quantified in Section 5.7.

Unlike the kinematic factors, this is a full quadratic attractor rather than a hinge, active everywhere. Its covariance $\sum_{\mathrm{lane}}$ governs a central trade-off: a tight setting enforces strict lane keeping but resists the displacement needed to avoid a lead vehicle, whereas a loose setting grants the avoidance factor freedom but risks lane departure. This competition is resolved through the relative weighting of the lane-centerline and avoidance factors (Section 4.4). Lateral containment within the corridor is provided complementarily by the obstacle factor (Section 4.4), which penalizes proximity to the corridor boundary and so discourages departure even when the centerline attractor is relaxed for avoidance.

### 4.4. Surrounding-Vehicle Interaction Factors

Safe navigation requires the trajectory to react to surrounding vehicles and stay clear of static obstacles. In factor-graph motion planning, static obstacles are handled by a hinge on a precomputed signed-distance field ϕ(·) (an idea introduced in CHOMP [56] and expressed as a factor in the continuous-time formulation [59]), with residual $r_{\mathrm{obs}}\left(x_{k}\right)=\max\left(0,\epsilon -\phi \left(x_{k}\right)\right)$. For static clearance, the present planner uses such a signed-distance-field factor and adds two lightweight interaction factors operating on the predicted relative geometry between ego and detected vehicles. The three are complementary: the SDF factor gives dense geometric clearance from the static environment and corridor and the interaction factors a prediction-based response to moving vehicles.

#### 4.4.1. Signed-Distance-Field Obstacle Factor

The signed-distance-field obstacle factor penalizes support states whose distance to the nearest obstacle falls below a safety margin ϵ. With the vehicle body approximated by a small set of bounding spheres of radius $r_{b}$ centered at $p_{b}\left(x_{k}\right)$, the residual is

(19) $$f_{\mathrm{sdf}}\left(x_{k}\right)=\sum_{b}{∥\;\max\;\left(0,\;\epsilon +r_{b}-\phi \left(p_{b}\left(x_{k}\right)\right)\right)\;∥}_{\sigma_{\mathrm{sdf}}}^{2},$$

where ϕ(·) is the signed distance to the nearest occupied cell in a locally built field and ∇ϕ supplies the Jacobian. The factor is formulated as a unary Pose2 factor with residual and Jacobian derived in the SE(2) tangent space, as for the lane-centerline and avoidance factors, so its information contribution is dimensionally consistent and linearizes cleanly. Queried over the corridor, it also acts as a soft lane-boundary term, inactive inside the corridor and growing near the boundary, complementing the centerline attractor. The dynamic response to moving vehicles, for which a static field is ill-suited, is delegated to the two interaction factors below. The same SDF factor, with the field precomputed offline, appears in recent factor-graph racing planners [67].

Each detected vehicle has position $\left(o_{x},o_{y}\right)$ and relative velocity $\left(o_{vx},o_{vy}\right)$ in the ego frame and a safety radius $r_{\mathrm{safe}}$ from its bounding box plus a margin. Under constant velocity its predicted position at support state *k* is $o_{k}=\left(o_{x}+o_{vx}\;t_{k},\;o_{y}+o_{vy}\;t_{k}\right)$ with $t_{k}=k\;\Delta t$.

#### 4.4.2. Lateral-Avoidance Factor

The lateral-avoidance factor penalizes support states intruding into a lead vehicle’s safety region, encouraging lateral deviation where free space exists. To restrict this to same-lane vehicles, a lateral gate $d_{\mathrm{gate}}$ ignores vehicles beyond the gate or behind the ego. In hinge form,

(20) $$f_{\mathrm{avoid}}\left(x_{k}\right)={∥\;\max\;\left(0,\;r_{\mathrm{safe}}-d_{k}\right)\;∥}_{\sigma_{\mathrm{avoid}}}^{2},\;d_{k}=∥p\left(x_{k}\right)-o_{k}∥,$$

which is active only when the vehicle lies within the lateral gate and ahead of the ego ($|d_{\mathrm{lat},k}|\leq d_{\mathrm{gate}}$ and $d_{\mathrm{lon},k}>0$), where $d_{\mathrm{lon},k}$ and $d_{\mathrm{lat},k}$ are the offset components in the path frame. The gate is essential: without it, adjacent-lane vehicles would be treated as obstacles, causing spurious avoidance and lane departures. Reacting to predicted relative geometry places this factor within interaction-aware planning [66,78], here reduced to a lightweight geometric term rather than a learned model.

The constant-velocity prediction aligns each state with the obstacle position at the matching time, so the factor reasons about future rather than instantaneous proximity and deviation begins before the encounter. Being time-indexed, its effectiveness depends on the support-state resolution: at highway speed the spacing can reach several meters, so a very near obstacle may fall between two states and engage only the immovable boundary state. Avoidance is thus most effective at moderate distances, where several mutable states lie within the safety region, while nearer obstacles are handled by deceleration through the following factor.

#### 4.4.3. Longitudinal-Following Factor

When the ego cannot deviate laterally, it must regulate speed to keep a safe gap. Inspired by adaptive cruise control and classical car-following such as the Intelligent Driver Model [79] and ISO 15622 [80], the longitudinal-following factor bounds speed by the available gap. With $d_{\mathrm{lon},k}$ the longitudinal distance to the lead, $d_{0}$ the standstill distance, and τ the time headway,

(21) $$v_{\mathrm{adm},k}=\frac{d_{\mathrm{lon},k}-d_{0}}{\tau },\;f_{\mathrm{follow}}\left(\xi_{k}\right)={∥\;\max\;\left(0,\;s_{k}-v_{\mathrm{adm},k}\right)\;∥}_{\sigma_{\mathrm{follow}}}^{2},$$

likewise gated to same-lane leads. It shares the hinge structure of the speed-cap factor’s limit policy of Equation (22), silent below the admissible speed and quadratic above it, inducing smooth deceleration. The two differ only in the source of the bound, a dynamic gap here versus a static map attribute there.

#### 4.4.4. Complementary Behavior

The two factors jointly realize a human-like reaction to a slower lead: lateral avoidance when adjacent space exists, deceleration when it does not, their shared gate excluding adjacent-lane traffic. Neither encodes an explicit choice rule. The choice emerges from the optimization. Each is a hinge contributing cost only when violated, so with free lateral space the avoidance factor is cheaply satisfied by a small displacement, whereas when the adjacent lane is occupied or lane-keeping is stiff that displacement is expensive and the optimizer instead reduces speed. The relative weighting of $\sigma_{\mathrm{avoid}}$ and $\sigma_{\mathrm{follow}}$ thus acts as a continuous knob between an evasive and a cautious style. These are balanced against lane-keeping so the aggregate residual stays within the solver’s range: an over-stiff interaction factor inflates the initialization error until the linearized update, dominated by that residual, leaves the initialization essentially unchanged, a complete failure to react. Each interaction and speed-cap factor supplies its residual Jacobian to the solver in closed form, expressed in the Pose2 body tangent space; being scalar functions of the SE(2) pose and velocity, these follow directly from the chain rule, with the SDF factor reusing the standard field gradient ∇ϕ [59] reprojected into the body frame. All obstacle and interaction factors are attached to the support states. The GP interpolation of Section 4.1.3 densifies only the output trajectory handed to the controller, not the in-optimization collision check. At the run-time discretization, the support-state separation is vΔt, about 2.0m at a typical 12m/s cruise and about 3.7m at the 22m/s speed cap, which stays below the surrounding-vehicle safety radius $r_{\mathrm{safe}}$, so no between-node tunneling occurs in the tested speed range. At genuinely higher speeds the interpolated (binary) obstacle factor of standard GP motion planning [59] can be re-enabled to densify the check between nodes.

### 4.5. Speed-Cap Factor

A single *speed-cap* factor, F10, bounds the longitudinal speed of each support state. It is a hinge on the forward speed that stays silent while the speed is admissible and grows quadratically once the speed exceeds the bound $v_{\mathrm{cap},k}$, inducing a smooth deceleration toward that bound. It is one factor, not three: the hinge $\max(0,\;s_{k}-v_{\mathrm{cap},k})$ and its velocity Jacobian are the same in every case, and only the source of the bound $v_{\mathrm{cap},k}$ changes. Three sources feed it: a routine limit read from the map, and two semantic directives, stop and yield, that are the images of inferred behaviors. Each supplies a per-state bound to the identical hinge. When several are active at once, the tightest one governs, so no separate priority logic is needed.

The map limit is a quantity the geometric planner can read for itself, but the other two are not. The behaviors inferred in Section 3 have no such geometry: a red light has nothing to avoid, and a crossing pedestrian is discarded by the vehicle filter that gates the interaction factors, its bounding box falling below the minimum vehicle extent. Only the semantic layer knows the ego must stop or yield, so the stop and yield directives are the point where the symbolic and numerical halves meet. Their interface is deliberately narrow: only conclusions bounding the longitudinal speed at a place reach the trajectory layer. A stop directive carries a stop point $s=(s_{x},s_{y})$ and asks the ego to halt before it; a yield directive carries a zone center $z=(z_{x},z_{y})$, radius *r*, and speed cap $v_{\mathrm{cap}}$, and asks the ego to pass slowly rather than halt. Which inferred rule produces which directive is the grounding of Section 3.3 (Table 4). The remaining conclusions do not reach this interface: mustAvoid goes to the mission planner of Section 3.4 and needsReroute triggers a mission replan at the run-time coupling of Section 3.5, while same-lane car-following is handled geometrically by the longitudinal-following factor of Section 4.4 from the tracked lead, without a symbolic conclusion. Directives are issued in the map frame and transformed into the vehicle-local frame before assembly, by the same transformation as the goal and centerline hint.

The routine bound is the map limit. Each link carries a legal or contextual speed limit $v_{\max}$, which in the absence of any directive bounds the longitudinal speed of each support state in hinge form:

(22) $$f_{\mathrm{spd}}\left(v_{k}\right)={∥\;\max\;\left(0,\;s_{k}-v_{\max,k}\right)\;∥}_{\sigma_{\mathrm{spd}}}^{2}.$$

It is unary on the velocity, silent at or below the limit and growing quadratically above it. The per-state bound is $v_{\max,k}=\min\;\left(v_{\max}^{\mathrm{map}},\;v_{\mathrm{curve},k}\right)$, combining the link’s speed limit with a curvature-adaptive cap $v_{\mathrm{curve},k}=\sqrt{a_{\mathrm{lat}}^{\max}/\kappa_{k}}$ that lowers the speed on curves toward the lateral-acceleration comfort target $a_{\mathrm{lat}}^{\max}$. As a soft, one-sided penalty, it reduces rather than strictly bounds cornering lateral acceleration (Section 5.8), and in the default configuration, it is the sole cornering-comfort mechanism. Each link’s speed limit is assigned from its road type during the offline conversion of Section 5.1.3 and read directly as the per-cycle $v_{\max}^{\mathrm{map}}$, with no inference rule involved.

A stop directive replaces that bound with one that tightens as the stop point nears. With the longitudinal distance from state *k* to the stop point along the heading, $d_{\mathrm{lon},k}=\left(s_{x}-x_{k}\right)\cos\theta_{k}+\left(s_{y}-y_{k}\right)\sin\theta_{k}$, constant deceleration $a_{\max}$ and $v^{2}=2ad$ give the admissible speed $v_{\mathrm{stop},k}=\sqrt{2\;a_{\max}\;\max\left(0,\;d_{\mathrm{lon},k}\right)}$, and the factor penalizes any excess in hinge form:

(23) $$f_{\mathrm{stop}}\left(\xi_{k}\right)={∥\;\max\;\left(0,\;s_{k}-v_{\mathrm{stop},k}\right)\;∥}_{\sigma_{\mathrm{stop}}}^{2}.$$

The clamp inside the root makes $v_{\mathrm{stop},k}=0$ once the state has passed the stop point, driving those states to rest rather than releasing them. Attaching the factor to every state yields an emergent braking profile: distant states keep a high admissible speed, near states are bound to progressively lower speeds, and the GP prior smooths the sequence into a feasible ramp.

Unlike the map-limit and yield sources, which bound the velocity only, the stop bound depends on the pose through $d_{\mathrm{lon},k}$, so the stop source alone also contributes a nonzero pose block; its closed-form Jacobian vanishes outside the active region and steepens as the stop point nears, mirroring the braking profile itself.

A yield directive instead caps the speed only inside a disc. With the gate

(24) $$I_{\mathrm{zone},k}=I\;\left[\;∥p\left(x_{k}\right)-z∥<r\;\right],$$

the residual is

(25) $$f_{\mathrm{yield}}\left(\xi_{k}\right)={∥\;I_{\mathrm{zone},k}\cdot \max\;\left(0,\;s_{k}-v_{\mathrm{cap}}\right)\;∥}_{\sigma_{\mathrm{yield}}}^{2}.$$

The gate is a fixed indicator at the linearization point, so the pose Jacobian is zero and the velocity Jacobian is the shared forward-speed block [1, 0, 0]. The factor is a pure speed bound whose support is set by geometry. Neglecting the gate derivative is the same short-horizon approximation used for the following factor’s heading, and it keeps the residual piecewise smooth: states outside contribute nothing, states inside contribute the same hinge as the map limit. The two directives differ only in profile shape, mirroring the two directive forms in the rule base: the stop bound varies with distance and so decays to zero at the line, whereas the yield bound is constant within the zone and so is flat at $v_{\mathrm{cap}}$ and recovers afterward. A crossing pedestrian and a merging vehicle thus produce qualitatively different trajectories, and the difference originates in the rule that fired, not in optimizer tuning.

Because the three sources feed one factor, arbitrating a routine limit, an inferred stop, and an inferred yield needs no separate priority logic. Together with the longitudinal-following factor of Equation (21), which shares the same hinge form, their bounds span the planner’s speed sources (Table 6).

This uniformity is what yields the arbitration without explicit priority logic, and the stop and yield speed profiles it produces are exercised in Section 5.6. Several concurrent recommendations each become a hinge on the same forward speed $s_{k}$. Since the cost sums the active hinges, the most restrictive bound dominates at the optimum and the looser ones are effectively silent. Because the hinges are soft, the optimum settles slightly above the tightest bound rather than exactly on it, by an amount set by the stiffness ratio to the prior. A cycle in which a stop directive and the geometric following bound are both active thus collapses toward the stop profile, not by a written precedence but because the stop-line hinge is the most restrictive. The rule base thus stays declarative while the trajectory layer resolves the conflict within the same least-squares problem.

#### Stiffness and Solver Interaction

The covariances $\sigma_{\mathrm{stop}}$ and $\sigma_{\mathrm{yield}}$ need the same care as the interaction stiffnesses, for the same reason. A stop directive issued at cruising speed places a large residual on every state at once, since the admissible speed is far below the current one along the whole horizon. If $\sigma_{\mathrm{stop}}$ is too tight, the initialization error exceeds the solver’s acceptance range and the incremental solver returns the initialization unchanged, the same non-reaction failure as in Section 4.4. Loosening it restores convergence while still bending the profile toward the bound before the stop point. Concrete values and the resulting profiles at both settings are reported in Section 5.

### 4.6. Semantic-Aware Global Replanning

The interaction factors act on a single local trajectory and so are confined to within-lane maneuvers. When a lead is persistently slower, a lane change is the right response, a routing- rather than trajectory-level decision. The planner closes the loop with the global router by projecting blocking vehicles onto the semantic map and elevating the affected links’ routing cost.

For each detected vehicle ahead within a trigger distance, in the same lane, and slower than the ego, the corresponding link is found by mapping the predicted position onto the map. To avoid oscillatory routing, a hysteresis marks a link blocked only after $n_{\mathrm{confirm}}$ consecutive occupied cycles and releases it after $n_{\mathrm{release}}$ unobserved cycles. With B the set of dynamically blocked links, the routing cost is

(26) $$C\left(\ell \right)=C_{\mathrm{tosm}}\left(\ell \right)+w_{\mathrm{blk}}\;I\;\left[\ell \in B\cup B_{\mathrm{static}}\right],$$

where $C_{\mathrm{tosm}}\left(\ell \right)$ is the TOSM implicit-attribute cost, $B_{\mathrm{static}}$ the statically restricted links, and $w_{\mathrm{blk}}$ a large penalty. Whenever B changes, the graph is rebuilt and the route recomputed toward the unchanged goal. The elevated cost makes the router select an adjacent lane when a feasible change exists, and the resulting centerline becomes the new hint. A single semantic mechanism thus produces a coherent three-level response to a slower lead: in-lane lateral avoidance, longitudinal deceleration, and, when warranted, a lane-change detour. This response spans the local interaction factors and the global routing layer.

The lane change here is driven by the dynamic slow-lead cost. A second, symbolic source of lane preference is the prefersLane conclusion of rules B-4/B-6, which is folded into the mission-route cost. The router routes through a preferred lane when the resulting detour stays within a bounded overhead ($\kappa_{\mathrm{pref}}$ hops, two in our configuration) of the shortest route and the path remains connected; otherwise, it keeps the shortest route. This is equivalent to placing a large routing-cost bonus on the preferred lane, $C\left(\ell \right)\;-\;w_{\mathrm{pref}}\;I\left[\ell \in \mathrm{prefersLane}\right]$, capped by the overhead bound so the preference never forces an unbounded or disconnected detour, and it composes with the per-lane-change penalty $w_{\mathrm{lane}}$ and the dynamic cost of Equation (26). Whichever source commits to the change, it reaches the trajectory identically, as a shift of the routed centerline that the lane factor F7 tracks. The realization of prefersLane in the mission route is quantified in Section 5.2.3.

#### 4.6.1. Route Source

The routing above reacts to what the geometric front-end observes: a slower vehicle is projected onto a link, its cost raised, and the router re-solves. This must be separated from a second, independent routing source, because the two act on different evidence and the distinction matters for what the system decides.

The mission planner of Section 3 returns a plan whose drive actions name a lane sequence, and that sequence is the route. When a mission plan is available, the global planner runs no graph search: the named lanes are resolved to links, their centerlines concatenated by the same procedure that would consume a search output, and the polyline becomes the reference hint. Because a mission route, when used, replaces the graph search rather than layering on top of it, the two are alternative route sources selected by mode, not two costs arbitrated within one search. In the deployed default, the geometric router of this section owns the route, so the slow-lead cost of Equation (26) produces the lane-change detour while the mission plan contributes its ordered handling directives (stop, yield) rather than the lane sequence. When the semantic-route mode is instead enabled, the mission plan’s lanes are the route and the geometric dynamic cost is not consulted. In either mode only one source is authoritative at a time, so the symbolic *which-lanes* decision and the geometric *when-too-expensive* decision do not compete; the symbolic layer has in both cases already excluded the places it inferred unsafe.

Two safeguards keep the deferral honest. A lane sequence is accepted only if every identifier resolves to a loaded-map link. Otherwise, it is discarded and the router falls back to its own search, so a stale or malformed plan degrades to the geometric baseline rather than to no route. Each consecutive pair is also checked against the routing graph, and a pair that is neither a successor nor a permitted lateral neighbor is reported, since the symbolic lane graph and the routing graph come from different sources and need not agree. Retaining the geometric router as fallback also makes the two route sources a configuration choice, used in Section 5.

#### 4.6.2. Illustrative Three-Level Response

The three-stage slower-lead response is a property of the architecture rather than a dedicated module, demonstrated stage-by-stage on recorded data: the in-lane nudge under obstacle injection (Section 5.9), the deceleration-versus-lane-change transition on a single recorded segment under the reroute toggle (Section 5.10), and the routed lane change reproduced by a forced hint at the trajectory layer (Section 5.7).

### 4.7. Planner Configuration and Real-Time Operation

The planner’s behavior is set by a compact set of interpretable parameters, and its real-time viability rests on the sparse factor graph. This section consolidates those parameters and analyzes the structure enabling incremental replanning.

#### 4.7.1. Parameter Configuration

The planner’s parameters are organized by layer: discretization, GP prior, boundary, kinematic, comfort, lane, interaction, speed-cap and directive, and replanning. Apart from the lateral and longitudinal comfort limits, all are covariances and thresholds tuned once for the platform as relative stiffnesses rather than absolute gains, since every objective is a whitened residual (Section 4.2 and Section 4.4). Their symbols, meanings, and concrete values are consolidated in Section 5.1.6, as used in every experiment.

#### 4.7.2. Computational Structure and Complexity

Each support state $\xi_{k}=\left(x_{k},v_{k}\right)$ contributes six variables, so the problem has 6(N+1). As established in Section 4.1.4, only the binary GP prior and the ternary jerk terms couple distinct states, so the information matrix is banded with fixed bandwidth and one solve costs O(N) via sparse Cholesky rather than $O(N^{3})$, maintained incrementally by incremental smoothing and mapping [60,62]. Because the problem is re-centered into a new vehicle-local frame each cycle, the dominant saving is this banded O(N) Cholesky together with the warm start from the previous trajectory rather than cross-cycle reuse of the Bayes tree; the interpolation of Section 4.1.3 further decouples optimization resolution from output resolution, keeping *N* small without thinning the executed trajectory. Per-cycle run times are reported in Section 5.

## 5. Experiments and Results

This section evaluates the proposed framework along the two layers developed in the preceding sections: the semantic reasoning and mission planning of Section 3, and the factor-graph local trajectory optimizer of Section 4. Because the central contribution is the unification of these layers under a single TOSM representation, the evaluation substantiates two questions in turn. First, does the semantic layer produce mission routes that are (i) as good as standard graph search in path length (we make no separate routing-speed claim, Section 5.2), and (ii) able to act on inferred semantic constraints: avoiding inferred hazards, including mandatory waypoints, and ordering the handling actions that a router cannot express? Second, does the factor-graph optimizer produce feasible, smooth, lane-consistent trajectories that are competitive with, or better than, a diverse set of trajectory baselines, and does each factor contribute measurably to that outcome?

All experiments use the Busan semantic HD map and the recorded on-road logs described below. To keep each measured quantity unambiguous, the trajectory evaluation is reported in three complementary regimes (per-state, closed-loop drive-along, and a real-bag run of the deployed node), defined with their roles in Section 5.1.5.

### 5.1. Experimental Setup

This subsection describes the common basis of every experiment: the instrumented vehicle and the two sensor streams the planner consumes, the recorded Busan route and the semantic HD map built from it, the reasoning-generated evaluation route, the three evaluation regimes, and the concrete planner configuration held fixed throughout.

#### 5.1.1. Vehicle Platform and Sensor Configuration

**Figure 5:**
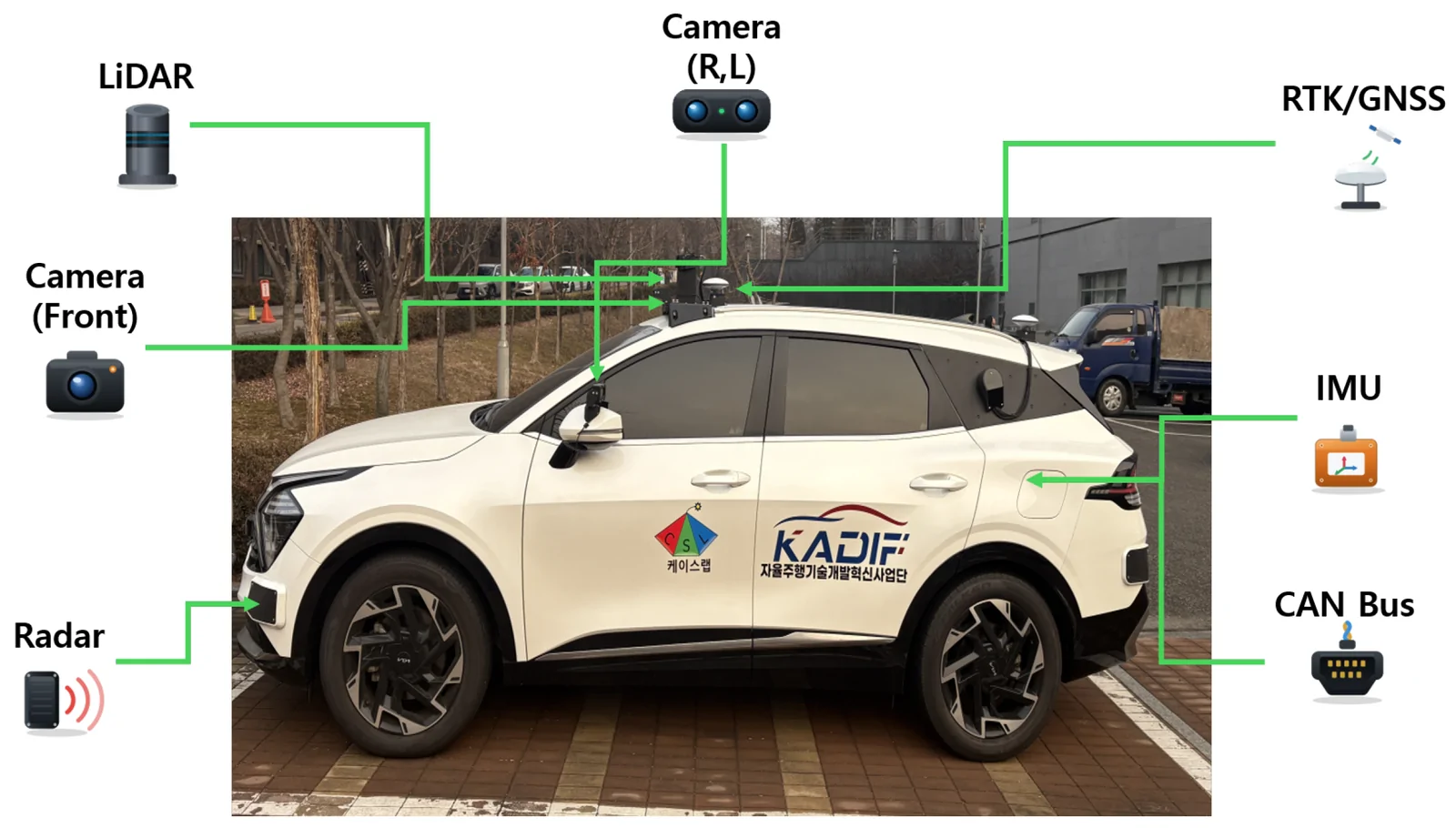
Instrumented test vehicle and the mounting positions of its sensors: roof-mounted LiDAR, left/right and front cameras, RTK/GNSS antenna, IMU, radar, and the CAN bus interface.

The data were collected with the instrumented vehicle of Figure 5, a Kia Sportage carrying a roof-mounted LiDAR, a camera, OEM radars, an IMU, and RTK/GPS, with on-board computation on an industrial PC. Although the platform is fully multimodal, the proposed planner consumes only two streams: the *LiDAR*, from which surrounding vehicles are detected and tracked by a standard front-end that supplies each vehicle’s position, velocity, and bounding-box extent (which sets the safety radius $r_{\mathrm{safe}}$) to the interaction factors, and the *RTK/IMU*, which supplies the ego pose used as the boundary state of the local trajectory optimizer. This detector is a stock component and is not itself evaluated: the reasoning route is obstacle-free, so the obstacle and interaction factors (F6, F8, F9) are inactive on it, F6 appearing dormant in the factor ablation (Section 5.4) and F8/F9 being exercised separately under synthetic obstacle injection (Section 5.9). The detector is a standard, independently developed front-end. Its uncertainty characterization is orthogonal to the reasoning-to-motion contribution and is handled at the perception interface (Section 3.2).

#### 5.1.2. Data Collection Route

**Figure 6:**
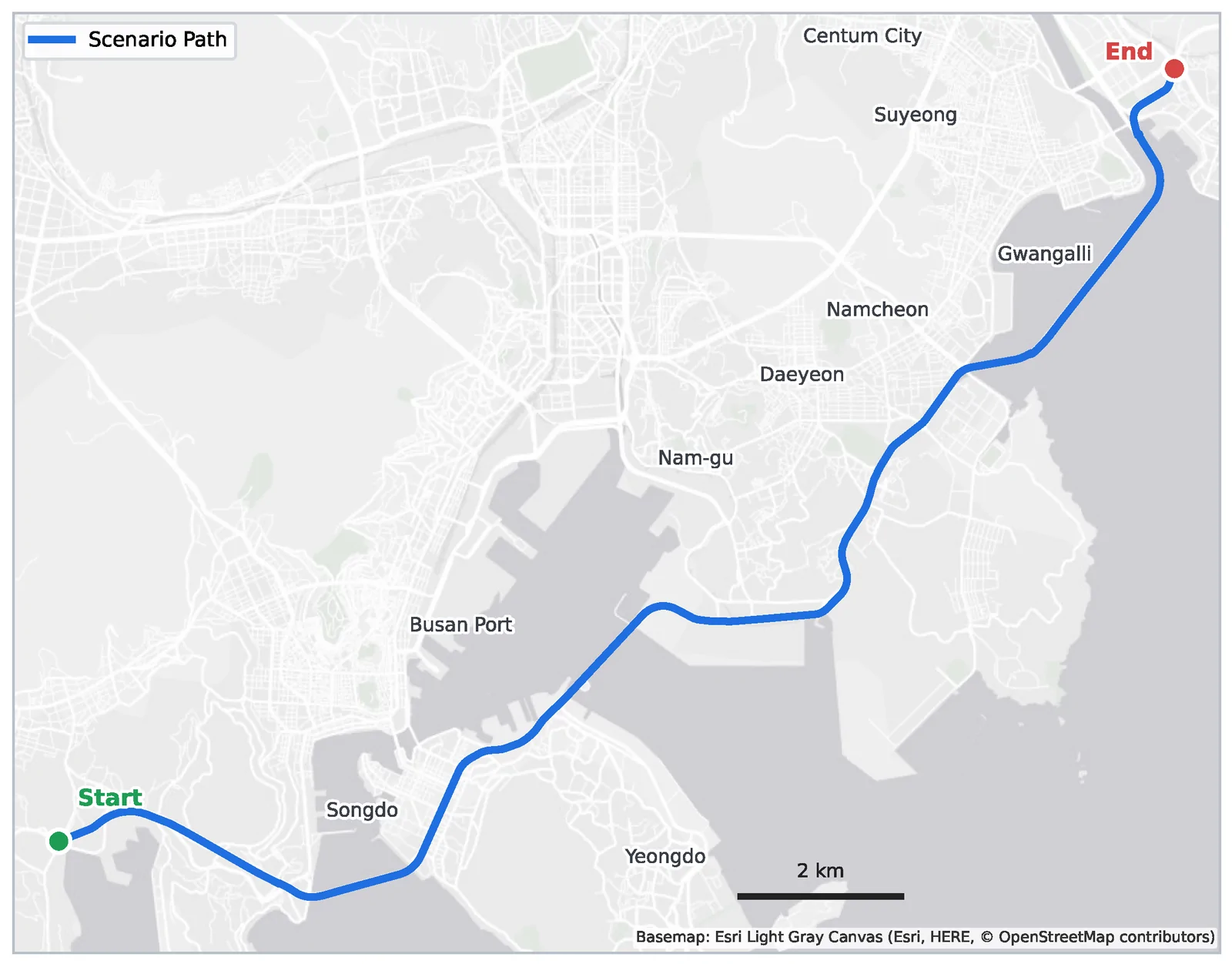
Data collection route in Busan.

The driving logs were recorded over a heterogeneous multi-segment route in Busan, Republic of Korea (Figure 6), traversing general urban roads together with several elevated bridges and tunnel/underpass sections. This heterogeneity is useful because it exercises the structural categories the map records: consistent with the place hierarchy of Table 2, every span is a *RoadSegment* that carries its structural road type (road, bridge, tunnel, or underpass) as an attribute rather than as a separate class. The route therefore exercises the semantic HD map on the same structural distinctions the reasoning layer relies upon. The trajectory-quality experiments (Section 5.3) run on the reasoning route, whose clean geometry is free of these GPS-shadowed spans, so planner properties are separated from input-pose degradation.

On the tunnel and underpass spans, the RTK signal is shadowed, so the georeferenced ego pose degrades. Because pose estimation under such conditions is the subject of the prior localization work [7] rather than of this paper, the present evaluation treats GPS-shadowed spans as a known limitation of the input pose and reports planner behavior on them accordingly, rather than as a claim about localization accuracy. These degraded-pose spikes originate in the input pose supplied by the localization subsystem under GNSS shadowing, not in the planner, which reflects its boundary state faithfully. The trajectory-layer smoothness terms bound how fast a corrupted boundary pose propagates into the executed path but cannot reconstruct a pose the localization layer did not provide, so this span defines the inherited operational-design-domain boundary of the present planner.

#### 5.1.3. Semantic HD Map Construction

The base geometric map for the route is obtained in the HD map format [28] and converted into the TOSM-based semantic HD map of Section 3 through the conversion procedure described there. Each drivable link becomes a *RoadSegment* individual (the source export’s legacy *Lane* label is folded into *RoadSegment* per Table 2, so “lane” below denotes such a drivable-link individual, not a separate class) nested in its enclosing road-segment group by isInsideOf, neighbor links are encoded as isLeftOf/isRightOf, and perceptible objects are attached as the corresponding *LandmarkObject* subclasses, yielding the reasoning-ready map used in all experiments below. Each lane also carries its surveyed speedLimit attribute from the source data (30–80km/h on this route). Consistent with the design of Section 3.3, this limit is not a rule conclusion: the deployed planner reads it directly from the ego lane and applies it as the map-limit policy of the speed-cap factor (F10). Where the survey value is missing, the speed limit falls back to the road-type default. Table 7 lists the HD-map attributes the semantic layer and planner actually consume, the value each takes on this route, its TOSM encoding, and the component that reads it. Raw survey fields not read downstream (internal link identifiers, boundary-marking codes) are omitted.

The conversion yields a consistent knowledge base (Table 7): the Pellet classifier reports no unsatisfiable class and the passability inferences (canMoveTo, isPassable) are produced without violation, so the map is faithfully represented before any behavioral reasoning is added. Of the 1086 links, 910 are nested inside a group by isInsideOf; the rest are free-standing, not grouped into an enclosing place by the source export.

Beyond static consistency, the fast forward-chaining engine that fires the behavior rules at run time was cross-validated against the Pellet description-logic reasoner. Both reasoners were given the same materialized qualitative predicates (including the closed-world isPassable=¬isBlocked, computed once at the perception interface), so the comparison is over the positive rule fragment. On nine scenarios that jointly exercise the stop, yield, lane-preference, and passability rules (B-1–B-5, P-1, P-2), the two reasoners produced identical behavioral inferences in every case (9/9), each Pellet solve taking about 1.3s. The run-time engine is therefore a faithful implementation of the declarative rule base rather than an approximation of it. These nine cross-validation scenarios are a subset of the eleven-scenario set used later for the rule ablation (Section 5.5) and PDDL ordering (Section 5.2.2); the two additional scenarios exercise the merge-side preference (B-6) and route-blocked reroute (E-1) rules, which is why cross-validation covers seven of the nine rules. Beyond this curated set, the same parity was run on 88 randomized reasoning scenarios, varied over the map templates by per-object time-to-collision band, signal color, and intention, and by per-lane congestion. The two reasoners agreed on 100% of the hazard-response directives (stop, yield, reroute). The only discrepancies are on the passability relations mustAvoid/prefersLane, and these trace to unspecified neighboring-lane annotation in the randomizer rather than to a rule-logic difference. This cross-check is a soundness test that the run-time engine faithfully implements the declarative rule base, not a statistical claim of behavioral completeness. Rule necessity is assessed separately by the rule ablation (Section 5.5).

#### 5.1.4. Evaluation Route

The reasoning layer generates the evaluation route: given a start and destination lane, the mission planner (Section 3) returns a connected lane sequence over the semantic map, which is expanded to a 2m-spaced centerline. On the Busan map this yields a 20.6km route of 81 lanes, used as the common global path for the trajectory experiments so that every planner is evaluated on the same reasoning-produced route. All six baselines and the proposed planner track this identical route. No method is given a geometry the others do not see.

#### 5.1.5. Evaluation Protocol

Following the taxonomy of planning benchmarks [81], the trajectory results are reported in three complementary regimes so that each measured quantity is unambiguous. The *per-state* regime scores the planned trajectory over all support states of a single query (the quantity the factor graph directly shapes) and is used for the factor ablation (Section 5.4). The *simulated closed-loop* (*drive-along*) regime executes the planned pose and feeds the resulting ego state back as the boundary state of the next query, so accumulated tracking error is exposed; it is closed-loop in the planning sense (the executor replays each planned pose and the planner re-solves from it) but does not actuate the vehicle (the actuated deployment is left to future work). It is used for the baseline comparison (Section 5.3). The *real-bag* regime runs the deployed ROS node on the recorded Busan drive with the reasoning route as its global path (Section 5.8), evaluating the planner on the real logged pose stream. The regimes are complementary: only the closed-loop regime ranks all seven methods, while the per-state ablation and the real-bag run confirm that ranking where they overlap.

#### 5.1.6. Deployed Planner Configuration

Table 8 lists the concrete values of the planner parameters defined symbolically in Section 4.7, as used in every experiment below. They were tuned once for the platform and held fixed across all routes and regimes. Every stiffness is reported as the standard deviation $\sigma =\sqrt{\sum }$ of its whitened residual, so a smaller value binds harder. The speed-limit value is only the fallback bound: in the deployed pipeline, the per-lane map limit of Section 5.1.3 overrides it.

### 5.2. Semantic Route Planning and Mission Feasibility

We first evaluate the mission-planning realization of the semantic layer over the connectivity graph, using 300 start–destination pairs sampled by breadth-first farthest-reachable selection (seed fixed for reproducibility). The routing graph takes the union of longitudinal succession isConnectedTo and lane-change adjacency isLeftOf/isRightOf. Of the 1086 lanes, 1031 have at least one outgoing edge, which exceeds the 885 isConnectedTo succession assertions of Table 7, and the rest are free-standing links with no outgoing edge. Across the 300 pairs the router returns a route for every pair at a length within 0.5% of the distance-optimal Dijkstra [8] route, so its route quality is on par with a standard shortest-path search. We make no claim of a routing-speed advantage: the realization is a plain breadth-first traversal, so its cost relative to Dijkstra, A* [9], or greedy best-first reflects the queue discipline rather than the reasoning. What the geometry-only searches cannot do is honor the inferred hazard and mission constraints or order their handling; that capability, produced by the reasoning and PDDL layer (Section 5.2.2), is the semantic layer’s distinguishing value and is evaluated in the two experiments below.

#### 5.2.1. Constraint Satisfaction: Hazard Reroute and Mission Waypoints

The value of the semantic layer is that it consumes inferred constraints. Two are tested. For *hazard avoidance*, a lane on the nominal route is marked impassable (a run-time hazard that makes isBlocked hold: an incident, or a restricted or heavily congested segment) and the router must avoid it. A red light or a crossing pedestrian is deliberately not such a cause; those are handled in place by a stop or yield (Section 5.2.2), not avoided. A static shortest-path plan, which is computed once and does not know the hazard, is the comparison. For *mission completion*, a reachable off-route lane is designated a mandatory waypoint that the route must traverse. Table 9 reports the outcome. The reasoning router never routes through a blocked hazard lane (0% violation), whereas the static plan traverses it in every case (100%). This 100%, however, is a static-versus-replanning gap, not a semantic-versus-geometric one: given the same inferred impassability, a classical replanning graph search (edge-removal Dijkstra) makes the identical avoid decision on every one of the tested pairs and achieves the same 0% violation, reroute set, and length overhead. What the semantic layer uniquely supplies is the inferred impassability itself (a geometry-only stack has no way to conclude that a lane is blocked by a crossing pedestrian or a red phase) and, for a hazard that lies on the only corridor, the ordered handling of the next subsection. When a detour exists, the reroute costs only +0.8% in length, and the 78.2% reroute-success rate is bounded by map connectivity: the remaining pairs block an articulation lane whose removal disconnects the graph, for which no router can produce a detour and both routers correctly return *no route* rather than violating the constraint. Mandatory waypoints are included in 100% of missions (a via-point decomposition that, again, a replanning graph search given the waypoint also achieves).

#### 5.2.2. Ordered Hazard Handling: PDDL and Graph Search

The routing above raises the cost of a hazardous lane so the router steers around it, which a cost-augmented graph search can also do. The mission planner of Section 3.4 adds a capability that graph search cannot express: it inserts *ordered handling actions*. A hazard on the only corridor cannot be avoided (a crossing pedestrian, a red light, an emergency stop) and must instead be handled before the ego drives through its place, exactly the ordering enforced by the drive precondition of Section 3.4, whereas a shortest-path router returns a lane sequence with no notion of a handling action.

We evaluate this on eleven semantic scenarios spanning normal driving, traffic lights, merges, cut-ins, emergency braking, crosswalks, multi-path missions, and a combined case. Each scenario is run through the reasoner and the PDDL mission planner (solved by Fast Downward), and compared against a shortest-path graph search over the same corridor. On the five scenarios whose hazard is on the driven corridor and therefore unavoidable, namely a red light (B-3), an emergency-brake critical object (B-2), a crosswalk and a multi-path pedestrian (B-1), and a combined red-light case, the PDDL plan places the required stop_for/yield_to before the corresponding drive (ordering satisfied 5/5), while the graph search, unable to represent a handling action at all, drives through unhandled (0/5). For the crosswalk case, the plan is

stop_for(ego, ped) → drive(ego, *ℓ*_0_, *ℓ*_1_) → drive(ego, *ℓ*_1_, *ℓ*_2_) → ⋯ → arrive

the stop preceding the departure from the pedestrian’s lane. The shortest-path router returns the identical lane list $\ell_{0},\;\ell_{1},\ldots$ but nothing that makes the ego stop. A manually specified finite-state machine could insert a stop or yield when told a hazard is on the corridor. The point is not that ordered handling is unprecedented but that the PDDL domain derives it from the drive precondition for any configuration of hazards, including the combined scenario in which several perceived hazards coexist and the domain still handles the on-corridor one in order, without a per-scenario script. Across all eleven scenarios, the planner solves 10 (the one exception is a fully blocked route, handled instead by the E-1 reroute trigger and mission-replan path of Section 3.5). The mean plan time is 0.11s, incurred only on a semantic event rather than every cycle (Section 3.5), which is the cost of the ordering guarantee that graph search cannot provide.

The mission-layer guarantee is separate from how a stop or yield is physically carried out: the actual slowing is produced every cycle by the speed-cap factor acting on the current directive (F10, Section 5.6), so the ego would brake for an on-corridor hazard from the reactive directive path even with no mission plan at all. What the PDDL ordering adds is therefore not the braking itself but three properties the per-cycle path cannot supply on its own: a plan whose hazard handling can be checked before the ego departs the place rather than trusted to fire in the moment, a symbolic safety net that still sequences the stop if a per-cycle directive is dropped or delayed, and an auditable trace that ties each executed maneuver back to the inferred hazard that required it. The mission layer thus contributes verifiability and accountability over the reactive layer rather than a second, redundant actuation path.

#### 5.2.3. Lane-Preference Realization

A third inferred constraint the router consumes is the semantic lane preference prefersLane (rules B-4/B-6), folded into the mission-route cost as the bounded preference of Section 4.6. Across the eleven semantic scenarios the preference fires in three: a congested-lane cut-in (B-4), a merge-side preference (B-5’s companion B-6), and the combined case. The router honors it in two of the three, routing through the preferred lane at a +1-hop overhead (within the $\kappa_{\mathrm{pref}}=2$ bound) and thereby realizing the inferred lane change in the mission route where a plain shortest-path search omits it. In the third (the combined case), the preferred lanes are unreachable toward the destination without crossing a lane the reasoner has already marked impassable (mustAvoid), so the router correctly declines the preference and keeps the feasible route rather than forcing a disconnected detour. The preference thus alters the route only when a bounded, connected detour exists; the resulting centerline is then tracked by the lane factor F7 exactly as the routed lane change of Section 5.7, so the same trajectory-layer mechanism executes it whether the lane choice originates from this symbolic preference or from the dynamic slow-lead cost.

### 5.3. Local Trajectory Quality: Baseline Comparison

The proposed optimizer is compared against a diverse baseline set spanning algorithm classes: the Original GPMP2 (GP prior and obstacle SDF only, i.e., our factors removed), a curvature-adaptive NMPC, a Werling Frenet sampler, and three standard path trackers: Pure Pursuit, Stanley, and DWA. All methods track the identical 20.6km reasoning route, and each is cited in Table 10. The Original GPMP2 row is a structural ablation, not a tuned rival (it carries no lane-tracking objective at all), so the large multipliers against it upper-bound the benefit of adding our factors rather than measuring a fair contest; the fair peer comparison for tracking is the geometric trackers. Trajectory quality is the 95th percentile of lateral acceleration and lateral jerk, the RMS centerline deviation, and the worst-case deviation. Speed is not held identical across methods (geometric trackers cruise at a set speed, the optimization planners self-select from curvature), so lateral-acceleration figures partly reflect speed and the comparison rests on the speed-robust measures below. The per-lane posted speed limits of Section 5.1.3 act only in the deployed live pipeline (Section 5.8), not in this controlled comparison.

#### 5.3.1. Closed-Loop Drive-Along Comparison

**Figure 7:**
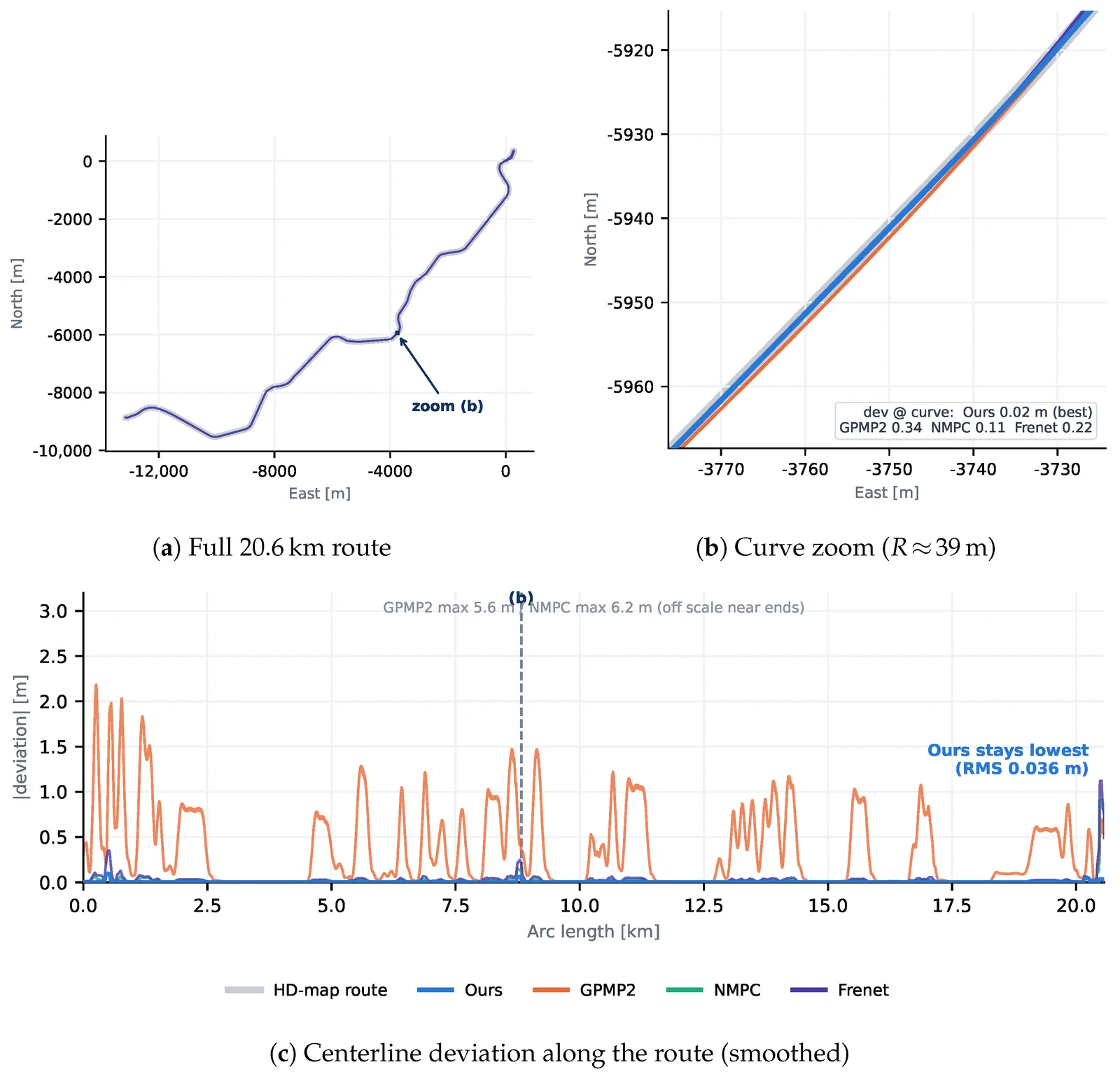
Executed trajectories on the reasoning route (blue: ours, orange: GPMP2, green: NMPC, purple: Frenet, gray: HD-map): (**a**) full route, (**b**) curve zoom, (**c**) centerline-deviation profile.

Table 10 reports the executed-trajectory comparison across the seven methods. Each plan is driven pose-by-pose with feedback and deviation is measured to the nearest point of the route polyline. All methods complete the route. The proposed planner has the tightest centerline tracking by a wide margin (0.036m RMS, a 7–26× improvement over the optimization baselines). Its tabulated worst-case deviation (1.59m) is a single sample at the near-stationary route terminus; the second-largest deviation over the whole drive is 1.06m, so in ordinary driving, its worst-case tracking stays below Pure Pursuit’s 1.55m, while the other five planners span 2.3–12.4m. Its lateral jerk is second only to the GPMP2 baseline, which reaches that smoothness only by ignoring the route (its RMS is 26× larger). The residual spread is diagnostic of each class: Stanley’s error feedback oscillates in lateral jerk, DWA’s discrete velocity sampling produces a large longitudinal jerk ($61\;{m/s}^{3}$), and Frenet’s sample switching drifts widely at curves. Because the pure trackers cruise near 12m/s while the curvature-aware planners self-select ∼9.8m/s, lateral acceleration ($v^{2}\kappa$) partly reflects speed. The speed-robust measures (centerline RMS and worst-case deviation) are where the proposed planner dominates, and unlike the geometric trackers it also carries the semantic, obstacle, and kinematic factors. Figure 7 shows the executed paths on the route and at a representative curve, together with the centerline-deviation profile along it. To confirm this ranking is not an artifact of baseline tuning, each baseline’s primary tracking gain was swept and every trajectory scored with the same segment-RMS metric: Stanley (best case 3.5×) and the dynamic-window planner (best case 1.7×) never approach the proposed planner at any setting, and Pure Pursuit falls below it on RMS only at a look-ahead so tight that its lateral jerk is 5.5× and its lateral acceleration 1.6× larger, so no baseline setting dominates the proposed planner on tracking and comfort at once.

Beyond the single detailed route, the ranking is validated on 24 seed-sampled routes with a Holm-corrected Wilcoxon test (Section 5.3.2). The proposed planner is significantly tighter than every baseline (p<0.001).

#### 5.3.2. Statistical Validation Across Routes

The comparison above uses one long route for a detailed, per-segment reading. To test that the ranking is not an artifact of that route, we sample 24 additional routes on the Busan map by farthest-reachable selection from a fixed seed (2–7km each). Because this map encodes lane topology largely through lateral adjacency rather than longitudinal succession, each sampled route’s centerline is stitched orientation-consistently and any residual degenerate out-and-back sample (a heading reversal where two parallel lanes are spliced) is discarded, so every route is a drivable forward drive. We run all seven methods on each route in the closed-loop drive-along regime and score every run by the same segment-distance metric. Table 11 reports the mean ± std over routes and the completion rate (a run counts as complete if it tracks at least 80% of the route length). All seven methods complete the set almost in full (every method completes all 24 routes except NMPC, which misses one), so the ranking is driven by tracking quality rather than by which methods finish. The proposed planner has the lowest centerline RMS and worst-case deviation on the aggregate, and a paired Wilcoxon signed-rank test (Holm-corrected for the six comparisons) finds it significantly tighter than every baseline on the centerline RMS (p<0.001 in all six) and likewise on the worst-case deviation. The one measure it does not lead is lateral jerk, where only the GPMP2 ablation edges it (0.15 vs. $0.18\;{m/s}^{3}$), and only by tracking the route an order of magnitude less tightly (0.74 vs. 0.060m RMS).

The route dependence shows most clearly in the geometric and sampling trackers: Pure Pursuit and Frenet, which track the long reasoning route tightly (Table 10), loosen markedly on the shorter sampled routes (Pure Pursuit 0.061→0.63m, Frenet 0.556→1.59m centerline RMS), while the two factor-graph planners hold their tracking across both regimes. Both trackers carry a fixed look-ahead (respectively a fixed lateral-sampling horizon) tuned to the large-radius arterial geometry of the reasoning route; on the shorter, more intersection-dense sampled routes that same horizon cuts corners and switches samples, so the error that stays small on gentle geometry grows on tighter geometry.

### 5.4. Factor Ablation

To attribute the quality of Table 10 to individual factors, each factor is removed in turn from the full configuration and the planned trajectory is re-scored in the per-state regime (the regime is chosen deliberately: under closed-loop re-planning, single-factor removal is masked because the graph is re-solved every cycle). Table 12 reports the result and separates the factors into three groups.

Two factors are *always active* on nominal driving: the nonholonomic factor F4, whose removal doubles lateral acceleration and jerk, and the lane-centerline factor F7, whose removal nearly triples the centerline RMS. Two are *conditionally active* on sharp curves: F10’s curvature cap and the min-turn-radius factor F5, which do not move the $p_{95}$ but reduce the lateral-acceleration maximum on the 1% of hairpin states by up to 1.8×, an effect visible only in the maximum, not the percentile. The remaining factors in the table (F6 obstacle SDF, F10’s map-limit cap, and the jerk prior) are *dormant* on this obstacle-free, moderate-speed route, which is the correct behavior: a factor should not perturb the trajectory when its constraint is inactive. F10’s per-state bound is the minimum of its map-limit and curvature caps, so the two are ablated independently: the map-limit cap is dormant because the posted limit rarely binds on this route, while the curvature cap is the component that trims the hairpin maximum. F6 is dormant here for a further reason: the ablation field carries only an injected obstacle, not the map corridor boundary, so this row isolates F6’s obstacle-avoidance role; its soft corridor-clearance role in the deployed pipeline (Section 4.4) rarely binds, since F7 keeps the trajectory centered. The interaction factors F8/F9 are likewise inactive on this obstacle-free route and are exercised separately by obstacle injection (Section 5.9). A revealing trade-off appears for F7: removing it degrades tracking ×2.7 while barely changing the lateral percentile, the same tracking-versus-smoothness tension seen in the real-bag study below.

### 5.5. Rule Ablation

The factor ablation isolates each factor’s contribution to the trajectory. The same leave-one-out analysis applied to the reasoning layer isolates each rule’s contribution to behavior. Each of the nine rules is disabled in turn and the eleven semantic scenarios are re-run through the forward-chaining engine, comparing the inferred relations against the full rule base. Table 13 reports, for each rule, the scenarios it fires in and what is lost when it is removed.

Two properties stand out. First, no rule is redundant. Although three rules (B-1, B-2, B-3) all conclude requiresStopFor, removing any one is not masked by the others, because each fires on a distinct object that the others do not cover: a crossing pedestrian, a critical object, and a red light. Every rule is the sole producer of at least one required conclusion, so the nine-rule base is minimal, in contrast to the factor ablation, where several factors were dormant on the evaluation route. Second, the passability rule P-1 is *always active*: it supplies canMoveTo in all eleven scenarios and gates every drive action, the reasoning analog of the always-on nonholonomic factor, and removing it disables routing entirely. As an end-to-end check, disabling all behavior rules and keeping only P-1 drops the stop and yield directives from nine to zero across the scenario set (the six stop firings of B-1–B-3 and the three yield firings of B-5 in Table 13). Without the reasoning layer, the geometric planner receives no hazard directive at all, so the hazard-handling behavior originates entirely in the rules rather than in the geometry.

### 5.6. Semantic Directive Execution: Stop and Yield

**Figure 8:**
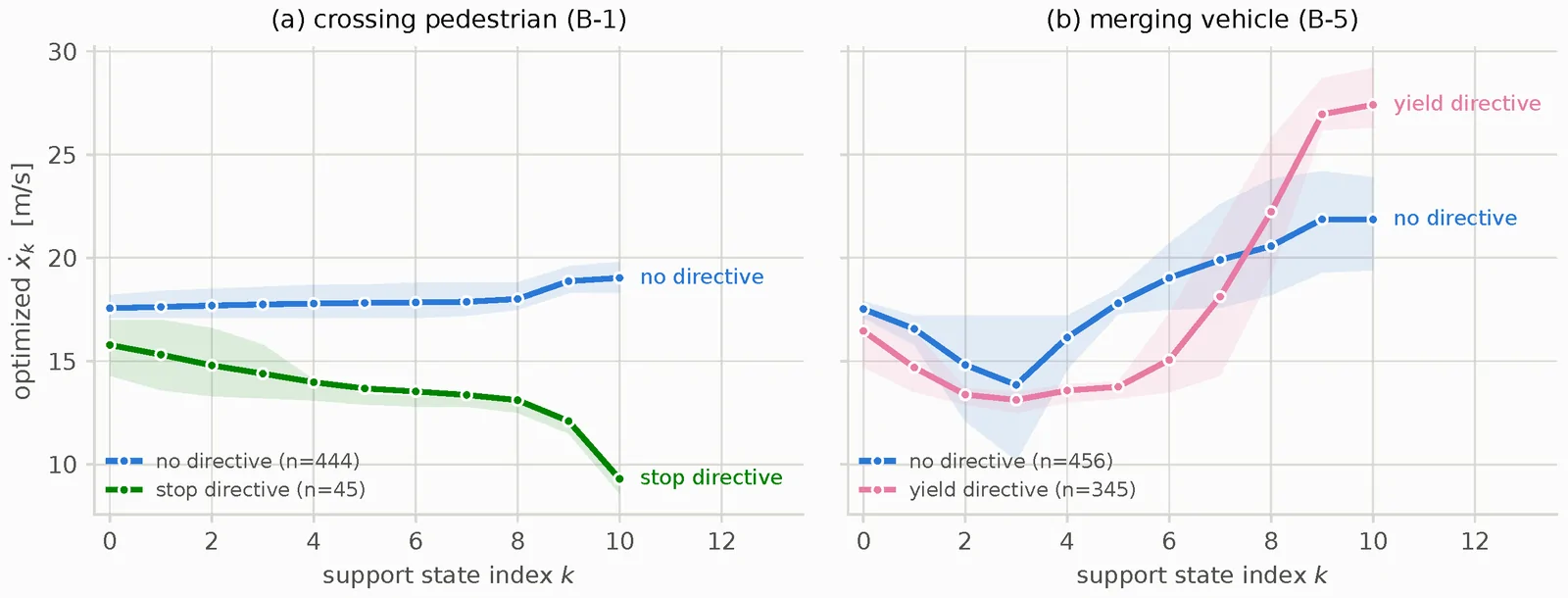
Optimized forward-speed profile ${\dot{x}}_{k}$ with a semantic directive active vs. matched no-directive cycles on the same approach: (**a**) a stop directive (B-1) yields a braking ramp; (**b**) a yield directive (B-5) caps speed inside the zone, recovering after.

The ablation above exercises the geometric and lane factors. This experiment tests the semantic directive factors of Section 4.5, the point where a symbolic conclusion becomes a trajectory-shaping force. On approach segments where the reasoner fires the pedestrian-stop rule (B-1) or the merge-yield rule (B-5) of Section 3.2, the resulting stop or yield directive is grounded into the speed-cap factor (F10) and the optimized forward-speed profile ${\dot{x}}_{k}$ is recorded across the support states. Because the LiDAR front-end supplies object geometry but not crossing intention or merge state, the qualitative predicates those two rules require (intention, isImminent, isApproaching) are supplied at the perception interface for this test, consistent with the interface assumption of Section 3.2. The trajectory response they induce is what is measured. Each panel of Figure 8 overlays the directive-active cycles against the matched no-directive cycles on the same approach, so the comparison is within one execution and not confounded by route or cruising speed.

The two directives produce qualitatively distinct profiles, exactly as the factor forms of Section 4.5 predict. The stop directive (Figure 8a) turns the flat ∼17m/s no-directive profile into a monotone braking ramp that decays toward the stop point (∼9m/s at the last state and continuing to rest), the distance-dependent stop bound of the speed-cap factor (Equation (23)). The yield directive (Figure 8b) instead depresses the speed to a roughly constant cap inside the zone (∼13m/s) and releases it afterward, the flat in-zone yield bound of the speed-cap factor (Equation (25)). After release, the profile briefly recovers slightly above the matched no-directive series before both settle to cruise, a jerk-limited settling transient, not a commanded overspeed: the post-zone speed-cap target returns to the posted limit (never higher), so the brief rise is only the smoothness-regularized optimizer re-accelerating to cruise from the lower in-zone speed. Because the shape of each response is fixed by which rule fired rather than by any per-case tuning, a pedestrian and a merging vehicle yield different trajectories through the same factor, confirming that the symbolic decision reaches the trajectory with its intent intact.

The same directive path also quantifies the debounce guard of Section 3.2 against perception false positives. In a longitudinal simulation of the directive-to-speed-cap path ($a_{\mathrm{brake}}=2.0\;m/s^{2}$ as above, 20 seeds), raising the per-frame false-positive rate from p=0.02 to p=0.20 without the guard (K=1) drives phantom-braking episodes from 7 to 57 per run with longitudinal-jerk spikes up to $35\;m/s^{3}$, confirming the brittleness a single-frame misfire can cause. A K=3 debounce suppresses phantom braking to near zero for p≤0.1 (and to 2.7 episodes per run at p=0.2), and K=5 removes it entirely for p≤0.1 and reduces it to 0.2 episodes per run at p=0.2, at a bounded detection latency of 0.2–0.4s (a few perception frames) and with every true hazard still detected. Even an unfiltered misfire remains bounded to the recoverable $a_{\mathrm{brake}}$ deceleration by the soft directive grounding, rather than a hard stop.

### 5.7. Semantic Lane-Change Execution

Lane changes are not decided by the local optimizer. However a lane change is chosen upstream, most directly when the semantic route crosses to a connected adjacent lane (Section 3), it reaches the trajectory layer the same way: the routed centerline hint shifts to the target lane, and the centerline factor F7 attracts the trajectory across while the kinematic factors keep it executable. This experiment therefore isolates the trajectory-layer question, whether a shift of the routed hint is executed as a comfortable, feasible lane change, by forcing the hint shift directly, independently of which upstream module produced it.

**Figure 9:**
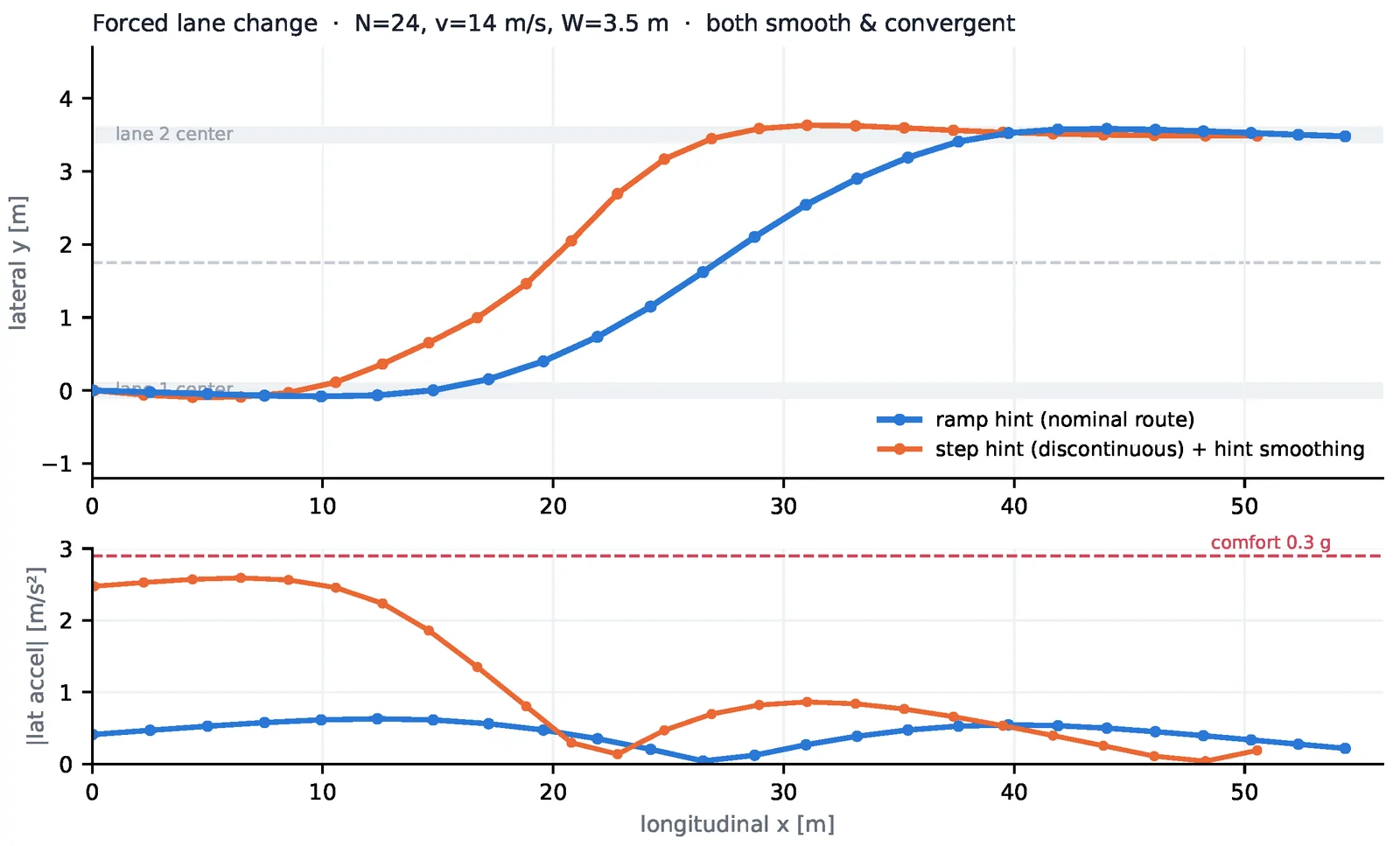
Forced lane change by shifting the F7 centerline hint (ramp vs. discontinuous step). In both panels, blue denotes the ramp hint and orange the step hint.

We reproduce a forced single-lane change (W=3.5m, entry speed 14m/s, 4s horizon) at the deployed discretization. Two hint shapes are compared: a *ramp* hint that models a nominal routed transition, and a *step* hint that models the worst case in which two adjacent lanelet centerlines are concatenated without overlap. Table 14 and Figure 9 report the result.

With the nominal ramp hint the change is completed over 37.6m at a peak lateral acceleration of $0.63\;{m/s}^{2}$ (about a fifth of the 0.3 g comfort reference) and a minimum turn radius of 307m. The discontinuous step hint is the stress case: at the chosen discretization the dense support states turn a zero-length lateral jump into a large local residual that, left untreated, diverges. An angle-gated hint pre-smoother, which relaxes only vertices whose turn exceeds a threshold and leaves nominal curvature untouched, removes the divergence, yielding a bounded, convergent change (peak $2.59\;{m/s}^{2}$) without affecting nominal tracking. The maneuver is thus executed by the same factor graph used for lane keeping, with no dedicated lane-change module: a shift of the routed centerline is realized as a smooth, feasible lane change, whatever upstream decision produced the shift.

### 5.8. Real-World Validation on Recorded Data

The preceding results use a drive-along executor. To confirm the ranking on the deployed system, the ROS node is run on the recorded Busan drive with the reasoning route supplied as its global path, which lets the optimizer plan continuously along the semantic route on the real logged pose stream. This is also the only regime in which the per-lane speed limits are live: as the ego traverses lanes, the reasoning layer feeds their surveyed limits to the speed-cap factor, which was verified to receive the correct posted value on every lane traversed in this window. The zones it crosses are 30/50/60/70km/h. The map defines six posted classes overall (30–80km/h); the 40 and 80km/h lanes present elsewhere on the map are not traversed in this replay window, so the speed-cap factor never receives their 11.1 or 22.2m/s bound here. The cap binds only where a lane’s limit falls below the free-driving speed. In a single replay at the deployed discretization (N=24), the full configuration and the GPMP2 baseline each plan 553 cycles on the same window, so the comparison shares the identical real-road input. Table 15 reports the outcome at highway speed ($\bar{v}\approx 20.6\;m/s$): the proposed planner tracks the route nearly seven times more tightly (0.043m vs. 0.295m mean deviation).

The worst-case deviation (3.95m) exceeds the lane width and so does not reflect ordinary in-lane tracking. The same real-bag run records input-derived extrema that no physical trajectory can produce (a logged longitudinal-acceleration maximum on the order of $10^{3}\;{m/s}^{2}$ and a longitudinal-jerk maximum on the order of $10^{4}\;{m/s}^{3}$), which can only arise from discontinuities in the input ego pose on the GPS-shadowed tunnel and underpass spans reported in Section 5.1.2. The centerline-deviation maximum is of the same origin: the degraded input pose, not the planner, dominates it, whereas the mean deviation (4.3cm) reflects the actual tracking.

The lateral acceleration is higher for the proposed planner ($p_{95}\;=\;5.9\;{m/s}^{2}$) than for GPMP2 ($3.0\;{m/s}^{2}$), but this is the cost of faithfully following a curved route at 74km/h: our planner tracks the curvature ($a_{\mathrm{lat}}=v^{2}\kappa$) while GPMP2 wanders off it and so averages less, at larger occasional spikes (maximum 9.4 vs. $5.2\;{m/s}^{2}$). Both exceed the 0.3 g target because the forward speed here is anchored to the logged ego velocity, so the curvature cap (a soft penalty) bends the profile toward the limit but cannot clamp it against the boundary state; a hard bound under a fixed velocity boundary is future work. Tracking, the speed-robust measure, is unambiguous. Longitudinal metrics from this pipeline are unreliable (a time-reconstruction artifact affecting both methods equally) and are not reported; the clean longitudinal comparison is the drive-along of Table 10.

### 5.9. Obstacle Handling

The reasoning route is obstacle-free, so the interaction factors are exercised separately, at the deployed N=24, by injecting static obstacles (150m spacing, 1.5m lateral offset, 2.0m safety radius) on a 5km segment of the reasoning route, which activate the lateral-avoidance and following factors (F8/F9). Table 16 compares the run with and without obstacles. Obstacle avoidance roughly doubles the mean solve time (2.1→4.4ms) while tracking and jerk are essentially preserved. On worst-case clearance the avoidance factor reduces the intrusion from −1.89m (GPMP2 baseline, no avoidance factor) to −0.58m, a 3.3× improvement, the residual reflecting that full clearance of a lane-intruding obstacle is a routing, not a local-smoothing, decision.

### 5.10. Three-Level Response on Recorded Data

The directive and obstacle experiments above exercised the interaction and speed-cap factors individually. This experiment exercises their composition into the graded three-level response of Section 4.6.2, by replaying one segment of the recorded Busan drive with a persistent slower lead in the ego lane and toggling the semantic-reroute mechanism of Section 4.6. In this configuration, the deployed geometric router owns the route, so the slow-lead cost of Equation (26) is the source of the lane-change stage.

The mildest response (a), an in-lane lateral nudge, is the lateral-avoidance behavior quantified under obstacle injection in Section 5.9; it does not arise in the centered-lead segment used here (the lead tracks the ego centerline, so no lateral offset reduces the intrusion), which isolates the deceleration and lane-change stages. The two outcomes are compared on the same route segment with the same lead vehicle. With reroute disabled, the trajectory stays in lane and the planner regulates speed behind the lead; the global route is never rebuilt and the blocked-lane detector never fires. With reroute enabled, the same lead is confirmed as a persistent in-lane obstacle, the routing cost of that lane is raised, the router rebuilds through the adjacent lane, and the local trajectory tracks the rerouted centerline into a lane change. Because the ego pose is fixed to the log, the experiment compares the maneuver the planner commits to under identical state, not the maneuver the vehicle executed. In the enabled run, the blocked-lane detector confirmed the persistent lead within $n_{\mathrm{confirm}}=3$ cycles. The hysteresis then held that lane in the blocked set for the remainder of the encounter (on the order of hundreds of cycles), keeping its routing cost elevated, while the route graph was rebuilt only on the transitions where the blocked set actually changed (not every cycle). In the disabled run, the detector never fired. The lane change is therefore produced entirely by the routing-cost mechanism of Equation (26), with no lane-change primitive in the optimizer, which simply tracks whatever centerline the router supplies.

### 5.11. Real-Time Performance

At the deployed discretization (N=24), the proposed planner solves incrementally in 2.1ms on average (5.1ms worst case), roughly 10× faster than re-solving the same graph from scratch with a batch Levenberg–Marquardt solve, and well inside a 20Hz replanning budget (a ∼25× margin). This incremental advantage, not any reduction in the factor set, is what incremental smoothing and mapping buys: the Original GPMP2 baseline, solved in its native from-scratch batch form, runs at 22.1ms per cycle (71.5ms worst case), the same order as a batch solve of our own graph and roughly 10× slower than our incremental update, while still tracking the lane poorly (Table 10). The factor-graph solve is faster than the NMPC prototype (5.7ms). The Frenet sampler is faster still (0.23ms) but only because it performs no optimization, at the cost of the worst lateral jerk in Table 10. These baseline prototypes are research implementations, so their absolute times are indicative only.

### 5.12. Closed-Loop Evaluation in Simulation

The experiments above are open-loop on recorded data. We add two closed-loop simulator evaluations that address two further questions. The first is real-time actuation, meaning the gap between replaying planned poses and executing them under a controller. The second is generalization to large-scale, standardized, and culturally diverse driving. Both evaluations use the same full pipeline (semantic HD map, run-time SWRL forward-chaining reasoning, and GP factor-graph optimization) with the same actuation the proposed method uses at run time (lane pure-pursuit steering with GP factor-graph speed and semantic behavior). We report the standardized-benchmark result in full and summarize the actuated-traffic result.

*Actuated closed-loop with dynamic traffic (CARLA).* We ran the proposed planner closed-loop in CARLA across three towns: a dense intersection town, a multi-lane town, and an urban town. The planner actuates the vehicle through Ackermann speed and steering, so the resulting pose feeds the next solve, and it runs with control latency and fifteen dynamic agents in the loop. Across twelve runs (three towns, four seeds), eleven completed without an at-fault collision, at a real-time cycle solve time of about 4ms and with lateral acceleration held below the 0.3 g comfort target throughout. The single failure is a boundary off-road excursion at one dense junction. It traces to the static drivable boundary not being wired into this trajectory-only test setup, not to the planner. This narrows the gap between pose replay and real-time actuation, although on-vehicle deployment remains future work.

*Standardized benchmark (nuPlan).* We ran the framework closed-loop on the nuPlan benchmark [81] across its four cities, namely Las Vegas, Boston, Pittsburgh, and Singapore, the last of which uses left-hand traffic. We used nuPlan’s standard two-stage (LQR) controller and the official closed-loop non-reactive score (CLS-NR). The run-time reasoner is the same SWRL forward-chaining engine the proposed method uses at run time, not a numeric surrogate. It materializes nuPlan detections into TOSM facts and fires the behavior rules online. To isolate the contribution of the semantic layer from the actuation, we compare against a geometry-only ablation (Original GPMP2) that removes the semantic factors and behaviors under identical actuation. As summarized in Table 17, the proposed planner improves CLS-NR by 1.53× across the four cities (0.686 vs. 0.447) and by 1.56× on the largest single-city subset (Las Vegas, n=58, 0.738 vs. 0.474), and it outperforms the ablation in every one of the four cities (Table 18). The gain concentrates in the safety terms. No at-fault collision, drivable-area compliance, and time-to-collision each improve by roughly 1.3–1.6×, which places the improvement in the semantic layer rather than in the shared actuation. The ablation is marginally higher only on comfort and progress, a by-product of over-aggressive driving without semantic speed regulation that comes at the cost of substantially more collisions. Absolute performance in the denser non-Vegas cities is lower. This is driven by three scenario types, namely lane-change, protected-turn-from-stop, and pedestrian-proximity, and, in the tightest intersection turns, by a residual actuation limitation. Even at the curvature-capped speed, the pure-pursuit tracker off-tracks by up to half a meter on the sharpest connectors, so a body corner briefly clips the drivable boundary where the human expert stays inside. Continuous in-optimization collision checking, controller-aware (tracker-in-the-loop) planning to remove this off-tracking, and a full val14 sweep are left as future work. We evaluate on the mini split with a representative scenario per standard type. The per-city figures for the smaller cities (Boston with n=7 and Singapore with n=4) are indicative, and the four-city aggregate is the statistically meaningful comparison.

### 5.13. Discussion

The experiments substantiate the unification claim from both ends. On the semantic side, the mission planner produces routes of standard-graph-search quality (within 0.5% of distance-optimal) and, at negligible cost, acts on inferred constraints a geometry-only stack cannot infer on its own: it never routes through an inferred hazard, always includes mandatory mission waypoints, and uniquely orders the required hazard-handling actions that a router cannot express (Section 5.2.2). On the trajectory side, across seven baselines spanning factor-graph, MPC, sampling, geometric-tracking, error-feedback, and dynamic-window classes, the proposed optimizer attains the tightest centerline tracking of all of them (1.7× over the strongest geometric tracker and 7–26× over the optimization-based baselines) at a near-lowest lateral jerk, while remaining real-time. The factor ablation attributes this to the nonholonomic and centerline factors on nominal driving and to the min-turn-radius and curve-speed factors on hairpins, with the remaining factors correctly dormant when their constraints are inactive. The closed-loop regime ranks the seven methods, and the per-state ablation and the deployed node on the recorded Busan drive confirm that ranking where they overlap, with the real-bag run confirming the tracking advantage at highway speed on real logged input. The one honest tension, that tighter tracking costs some lateral acceleration on curved high-speed segments, is a property of following the route rather than cutting across it, and is exactly the behavior a lane-consistent planner should exhibit.

## 6. Conclusions

This paper presented a semantic driving framework in which a single TOSM representation is carried from context-aware reasoning, through symbolic mission planning, into a continuous and kinematically feasible trajectory, closing the vertical gap between high-level semantic reasoning and low-level trajectory generation. A standardized HD map is lifted into a semantic HD map whose attributes describe every road element down to the per-lane posted speed limit and which is consumed, through a thin grounding that preserves its TOSM identities rather than re-modeling them, by both the reasoner and the trajectory optimizer. A set of SWRL rules infers context-appropriate maneuvers. An automatically generated PDDL problem, grounded directly in the inferred semantic state, sequences the inferred behaviors into a mission plan in which, by construction of the drive preconditions, every hazard on a place is handled before the ego departs it whenever the symbolic planner is used. A Gaussian-process factor graph turns that plan and the routed corridor into a smooth trajectory through semantic lane, interaction, and speed-cap factors, reusing the same incremental inference machinery as our prior localization work [7]. Because every layer reads and writes the same TOSM-grounded quantities, an inferred maneuver remains traceable into the trajectory that realizes it, so that the boundary between symbolic decision and continuous motion becomes an explicit, auditable interface rather than an opaque handoff.

The framework was validated on data from a real driving platform, with the behavioral predicates supplied at the perception interface. The semantic HD map formed a logically consistent knowledge base over which a description-logic reasoner found no unsatisfiable class, and the fast forward-chaining engine that executes the behavior rules agreed with that reasoner on every cross-validation scenario. The reasoning-driven mission router returned hazard-avoiding plans of near-optimal length, routed around inferred-impassable lanes at zero violation wherever a detour existed, realized the inferred lane preference prefersLane within a bounded, connected detour, and, through an automatically generated PDDL problem, inserted the required hazard-handling actions in the correct order on every on-corridor case. This ordered, hazard-aware plan is one that a static shortest-path baseline cannot produce, and the value lies in inferring the impassability and ordering the handling, not in the search itself. On the Busan route, the factor-graph optimizer tracked the routed lane markedly more tightly than a diverse set of trajectory baselines (by an order of magnitude over the optimization-based ones and more tightly than the strongest geometric tracker) and reproduced this advantage on recorded urban driving, all at a path curvature within the kinematic steering limit. Because the banded factor graph is re-solved incrementally rather than from scratch, the planner updated roughly an order of magnitude faster than a from-scratch batch solve, confirming its real-time viability. The graded response to a slower leading vehicle (in-lane avoidance, then deceleration, then a semantic-reroute lane change) follows by design from the interplay of the factors rather than from an explicit decision rule: on a recorded Busan segment with a persistent slower lead, toggling the semantic-reroute mechanism switched the committed trajectory between in-lane deceleration and a rerouted lane change under identical logged state. Each lane’s surveyed speed limit was applied at the speed-cap factor at the correct posted value per lane, though on the highway-speed recorded pass its enforcement was limited by the logged-velocity boundary state.

The present evaluation has several limitations. It was conducted open-loop on recorded driving data, the behavioral layer was checked for logical consistency and cross-reasoner agreement rather than by large-scale statistics, and the baselines are our own re-implementations. The unification is realized as a shared TOSM substrate with mode-selected route ownership, not a single controller in which every semantic source acts on one trajectory at once. Closed-loop deployment that actuates the vehicle, evaluation across additional urban environments, online HD-map maintenance and map-degraded operation, and robust pose estimation on GPS-shadowed spans remain as future work. More broadly, the results indicate that a single semantic substrate can hold the driving stack together across levels that are conventionally built on separate representations, a step toward autonomous driving that is at once capable and accountable. 

## Figures and Tables

**Table 1:** This work within our group’s TOSM line.

Work	Task Addressed	Layer on the TOSM Substrate		
		Behavior	Mission	Trajectory
Joo [4]	service-robot navigation	—	—	—
Bae [5]	multi-robot task allocation	—	task-level	—
Pyo [6]	object-centric pose	—	—	—
Choi [7]	vehicle localization	—	—	—
**This work**	on-road driving	**SWRL**	**PDDL**	**GP factors**

Bold indicates the present work.

**Table 2:** TOSM class hierarchy of environmental elements.

Class			
*EnvironmentalElement*	*Object*	RoadUser	Person
			Vehicle
			Bicycle
		LandmarkObject	TrafficLight
			TrafficSign
			SurfaceMark
			Facility
		UnknownObject	—
	*Place*	HDPlace	RoadSegment
			Intersection
		SemanticPlace	UnitPlace
		PedestrianPlace	Sidewalk
			Crosswalk
	*Robot*	EgoVehicle	—
	*Sensor*	Camera	—
		LiDAR	—
		Radar	—
		IMU	—
		GNSS	—

**Table 3:** Object properties of the semantic HD map.

Property	Domain	Range	Characteristics
*Spatial relations*			
isNextTo	Place/Object	Place/Object	
isInsideOf	Object/Place	Place	
isConnectedTo	Place	Place	
isLeftOf/isRightOf	RoadSegment	RoadSegment	inverse pair; ⊑ isAdjacentLane
isAdjacentLane	RoadSegment	RoadSegment	symmetric
*Robot and mission relations*			
isEquipped	Robot	Sensor	
isLocatedAt	Robot	Place	
hasDestination	Robot	Place	
isOnRoute	Robot	Place	
*Inferred relations*			
requiresStopFor	Robot	Object	
shouldYieldTo	Robot	RoadUser	
prefersLane	Robot	RoadSegment	
canMoveTo	Robot	Place	
mustAvoid	Robot	Place	

**Table 4:** Grounding of each inferred relation into the planner.

Inferred Relation	Grounded as	Consumed by
requiresStopFor	stop directive (with stop point)	speed cap F10, stop policy
shouldYieldTo	yield directive (speed cap)	speed cap F10, yield policy
prefersLane	lane preference (routing cost)	global router (Section 4.6)
needsReroute	mission-replan trigger	run-time coupling (Section 3.5)
canMoveTo	admissible successor place	mission planner
mustAvoid	forbidden place	mission planner
(map attribute)	speedLimit of ego link	speed cap F10, limit policy

**Table 5:** Factors of the local trajectory optimizer.

ID	Factor	Variables	Role
F1	Boundary (start)	$\xi_{0}$	Anchor start pose and velocity
F2	Boundary (goal)	$\xi_{N}$	Anchor look-ahead goal pose and velocity
F3	GP prior	$\xi_{k},\;\xi_{k+1}$	White-noise-on-acceleration smoothness
F4	Nonholonomic	$\xi_{k}$	Zero lateral body velocity
F5	Min. turn radius	$\xi_{k}$	Steering-geometry limit
F6	Obstacle (SDF)	$x_{k}$	Static/corridor clearance
F7	Lane centerline	$x_{k}$	Attraction to routed lane
F8	Lateral avoidance	$x_{k}$	Same-lane lateral evasion
F9	Long. following	$\xi_{k}$	Same-lane speed regulation
F10	Speed cap	$\xi_{k}$	Speed bound: map limit/stop/yield policy
F11	Longitudinal jerk	$v_{k-1:k+1}$	Comfort (fore-aft jerk) smoothness
F12	Angular jerk	$v_{k-1:k+1}$	Comfort (yaw jerk) smoothness

**Table 6:** Speed sources feeding the single speed-cap factor.

Factor	Bound	Source of the Bound
Speed limit, Equation (22)	$v_{\max}$	static map attribute
Long. following, Equation (21)	$v_{\mathrm{adm},k}$	measured dynamic gap
Stop line, Equation (23)	$v_{\mathrm{stop},k}$	inferred stop directive
Yield zone, Equation (25)	$v_{\mathrm{cap}}$	inferred yield directive

**Table 7:** Semantic HD map: (**a**) attributes consumed; (**b**) construction scale and consistency.

(a) Attribute	Busan Route	TOSM Encoding	Consumed by
Posted speed limit	30–80 km/h	speedLimit [m/s]	speed-cap F10 (limit)
Structural road type	Road/Bridge/Tunnel	*RoadSegment* attribute	place hierarchy, routing cost
Lane succession	succ./pred. links	isConnectedTo	mission planner, global router
Lateral neighbor	left/right link	isLeftOf/isRightOf	lane-change, replanning
Travel direction	one-way	one-way link	routing-graph direction
Lane centerline	2 m polyline	*RoadSegment* geometry	lane factor F7, route hint
**(b) Quantity**			**Value**
#Link/#Road-segment group/#Landmark			1086/622/369
#isInsideOf/#isConnectedTo/#isLeftOf+isRightOf			910/885/1156
Pellet consistency (unsatisfiable classes)			0
Reasoning time (Pellet, full map) [s]			1.27

**Table 8:** Planner parameter values used in all experiments (stiffnesses are σ; smaller binds harder).

Group	Symbol	Meaning	Value
Discretization	*N*/*T*/Δt	support intervals/horizon/step	24/4.0 s/0.17 s
GP prior	$Q_{c}$ (lon/lat)	translational PSD	1.0
	$Q_{c}$ (yaw)	rotational PSD	10.0
Kinematic	$\sigma_{\mathrm{nh}}$	nonholonomic (F4)	0.5 m/s
	$r_{\min}$/$\sigma_{\mathrm{turn}}$	min. radius (F5)	5.0 m/0.5
	$a_{\mathrm{lat}}^{\max}$	lateral-accel cap	0.3g
	$a_{\mathrm{lon}}^{\max}$	init pre-smoothing	$2.0\;m/s^{2}$
Comfort	$\sigma_{\mathrm{jerk}}^{\mathrm{lon}}$	long. jerk PSD (F11)	0.15
	$\sigma_{\mathrm{jerk}}^{\mathrm{yaw}}$	ang. jerk PSD (F12)	0.20
Lane	$\sum_{\mathrm{lane}}$	centerline attractor (F7)	0.4 m
Interaction	$\sigma_{\mathrm{sdf}}$/ϵ	SDF stiffness/margin (F6)	0.3/0.5 m
	$r_{\mathrm{safe}}$	bbox /2+ margin	+1.0 m
	$\sigma_{\mathrm{avoid}}$/$d_{\mathrm{gate}}$	avoid (F8)/gate	1.5 m/2.0 m
	$\sigma_{\mathrm{follow}}$/τ/$d_{0}$	follow (F9)/headway/standstill	1.5 m/s/1.5 s/5.0 m
Speed cap	$v_{\max}$/$\sigma_{\mathrm{spd}}$	fallback limit (F10)	22 m/s/1.0 m/s
Directive	$a_{\max}$/$\sigma_{\mathrm{stop}}$	stop braking/stiffness	$2.0\;m/s^{2}$/0.3 m/s
	*r*/$\sigma_{\mathrm{yield}}$	yield radius/stiffness	6.0 m/0.5 m/s
Hint smooth	kink/passes	angle gate/Laplacian	10°/25
Routing	$w_{\mathrm{lane}}$	lane-change cost weight	5.0
	$\kappa_{\mathrm{pref}}$	prefersLane detour bound	2 hops
	$w_{\mathrm{blk}}$	blocked-lane cost penalty	$10^{4}$
	$n_{\mathrm{confirm}}$/$n_{\mathrm{release}}$	block hysteresis confirm/release	3/10 cyc

**Table 9:** Semantic constraint satisfaction across the reasoning router, a replanning Dijkstra baseline, and a static shortest-path plan.

Metric	Ours (Reasoning)	Replan Dijkstra	Static Shortest
		(Given Signal)	
Hazard-lane violation rate	**0%**	**0%**	100%
Reroute success (detour exists)	78.2%	78.2%	—
Reroute length overhead	1.008×	1.008×	—
Mandatory-waypoint inclusion	**100%**	**100%**	—
On-corridor ordered handling	**5/5**	0/5	0/5
(stop-before-drive)			

Bold indicates the best value in each row.

**Table 10:** Closed-loop trajectory quality on the 20.6km route (95th-percentile, deviation to the route polyline; lower is better, best in bold).

Method	Centerline	Worst Dev.	Lat. Accel	Lat. Jerk	Class
	RMS [m]	[m]	[m/s^2^]	[m/s^3^]	
**Proposed (Ours)**	**0.036**	1.59	0.38	0.24	Factor graph (semantic)
Original GPMP2 [59]	0.942	5.63	0.35	0.15	Factor graph
NMPC [13]	0.238	6.24	0.71	1.94	Model-predictive
Frenet [77]	0.556	12.44	0.71	2.35	Sampling
Pure Pursuit [82]	0.061	**1.55**	0.51	0.30	Geometric
Stanley [83]	0.131	4.84	0.69	1.81	Error feedback
DWA [84]	0.064	2.26	0.63	3.08	Dynamic window

Bold indicates the proposed method and the best value in each column.

**Table 11:** Multi-route validation: mean ± std over 24 seed-sampled routes (closed-loop); *p* is the Holm-corrected Wilcoxon test on centerline RMS (best in bold).

Method	Complete	Centerline RMS [m]	Worst Dev. [m]	Lat. Jerk $p_{95}$	*p* (RMS)
**Ours**	24/24	**0.060 ± 0.015**	**1.26 ± 0.28**	0.18 ± 0.11	—
GPMP2	24/24	0.739 ± 0.411	3.07 ± 1.52	**0.15 ± 0.10**	<$10^{-3}$
NMPC	23/24	0.594 ± 0.125	6.20 ± 0.35	2.21 ± 1.07	<$10^{-3}$
Frenet	24/24	1.587 ± 0.215	11.64 ± 0.26	2.33 ± 0.79	<$10^{-3}$
Pure Pursuit	24/24	0.627 ± 0.065	7.62 ± 0.25	0.63 ± 0.50	<$10^{-3}$
Stanley	24/24	0.331 ± 0.040	4.28 ± 0.06	1.74 ± 0.93	<$10^{-3}$
DWA	24/24	0.640 ± 0.077	4.93 ± 0.32	3.29 ± 0.53	<$10^{-3}$

**Table 12:** Leave-one-out factor ablation (per-state, 20.6km route).

Configuration	Lat. Accel $p_{95}$	Lat. Accel Max	Centerline RMS	Effect
**full (Ours)**	**0.17**	5.19	**0.083**	baseline
−F4 nonholonomic	0.34	10.89	0.082	always-on
−F5 min-turn radius	0.17	5.80	0.084	hairpins
−F7 lane centerline	0.13	4.91	0.221	always-on
−F10 (map-limit cap)	0.17	5.38	0.083	dormant
−F10 (curvature cap)	0.17	9.52	0.081	hairpins
−jerk prior	0.17	5.19	0.083	dormant
−F6 obstacle SDF	0.17	5.19	0.083	obstacle-only

Bold indicates the full configuration used as the baseline for the ablation.

**Table 13:** Rule leave-one-out over the eleven scenarios: each rule disabled in turn, inferred relations compared to the full nine-rule base.

Rule	Inferred Relation	Fires in	Effect When Removed
P-1 Move to passable	canMoveTo	11/11	every mission loses its drive precondition (always-on)
B-1 Pedestrian stop	requiresStopFor	2	crossing-pedestrian stop lost
B-2 Critical-object stop	requiresStopFor	1	emergency stop lost
B-3 Red-light stop	requiresStopFor	3	red-light stop lost
B-4 Congested-lane pref.	prefersLane	2	lane preference lost
B-5 Merge yield	shouldYieldTo	3	merge yield lost
B-6 Merge-side pref.	prefersLane	1	side preference lost
E-1 Route-blocked reroute	needsReroute	1	reroute flag lost
P-2 Avoid blocked place	mustAvoid	3	avoidance lost

**Table 14:** Forced lane change (N=24). Comfort references: 0.3g ($\approx \;2.9\;{m/s}^{2}$) lateral acceleration, ∼$1\;{m/s}^{3}$ jerk [74].

Route Hint	Converged	Completion	Lat. Accel Max	Lat. Jerk Max	Min. Radius
		[m]	[m/s^2^]	[m/s^3^]	[m]
Ramp (nominal)	yes	37.6	**0.63**	0.98	307
Step (discontinuous)	yes	26.9	2.59	3.29	59

**Table 15:** Real-bag validation (N=24): deployed node on the recorded Busan drive, reasoning route as global path.

Metric	Ours	GPMP2 (Ablation)	Ratio
Centerline deviation, mean [m]	**0.043**	0.295	6.9×
Centerline deviation, max [m]	**3.95**	5.11	1.3×
Peak curvature κ [1/m]	0.025	0.017	follows road
Lateral accel, $p_{95}$ [m/s^2^]	5.85	3.00	see text

Bold indicates the better value of the two methods.

**Table 16:** Obstacle present vs. absent (Ours, N=24), same 5km route.

Condition	Conv.	Compute Mean/Max [ms]	Lat. Jerk Max	Min. Clearance [m]
Ours, no obstacle	99.5%	2.10/5.08	3.30	—
Ours, with obstacle	98.6%	4.42/26.0	4.23	−0.58
GPMP2, with obstacle	99.0%	—	38.7	−1.89

**Table 17:** nuPlan closed-loop CLS-NR score (best per column pair in bold).

Metric	4 Cities (n=88)		Las Vegas (n=58)	
	Ours	GPMP2	Ours	GPMP2
CLS (overall)	**0.686**	0.447	**0.738**	0.474
No at-fault collision	**0.886**	0.568	**0.914**	0.552
Drivable-area	**0.852**	0.670	**0.897**	0.724
Time-to-collision	**0.830**	0.511	**0.845**	0.500
Driving-direction	**1.000**	0.960	**1.000**	0.974
Speed-limit	**0.999**	0.994	**0.998**	0.991
Comfort	0.773	**0.830**	**0.862**	0.828
Progress	0.732	**0.938**	0.767	**0.937**

**Table 18:** Per-city nuPlan CLS-NR score. The ablation’s 0.000 in Boston reflects a score-zeroing violation (at-fault collision or drivable-area departure) in each of its seven scenarios, not a numerical error.

City	*n*	Ours	GPMP2
Las Vegas	58	**0.738**	0.474
Pittsburgh	19	**0.640**	0.577
Boston	7	**0.390**	0.000
Singapore	4	**0.666**	0.219
All 4 cities	88	**0.686**	0.447

Bold indicates the better value of the two methods.

## Data Availability

The data presented in this study are not publicly available due to project confidentiality agreements. Further inquiries can be directed to the corresponding author.

## Abbreviations

The following abbreviations are used in this manuscript:

CHOMP	Covariant Hamiltonian Optimization for Motion Planning
DL	Description Logic
GNSS	Global Navigation Satellite System
GP	Gaussian Process
GPMP	Gaussian Process Motion Planning
GPS	Global Positioning System
GTSAM	Georgia Tech Smoothing and Mapping
HD	High-Definition

IMU	Inertial Measurement Unit
iSAM	Incremental Smoothing and Mapping
ISO	International Organization for Standardization
LiDAR	Light Detection and Ranging
MAP	Maximum a Posteriori
OEM	Original Equipment Manufacturer
OWL	Web Ontology Language
PDDL	Planning Domain Definition Language
PSD	Power Spectral Density
RMS	Root Mean Square
RTK	Real-Time Kinematic
SDF	Signed-Distance Field
SE(2)	Special Euclidean Group in 2D
SLAM	Simultaneous Localization and Mapping
SMF	Semantic Modeling Framework
STRIPS	Stanford Research Institute Problem Solver
SWRL	Semantic Web Rule Language
TOSM	Triplet Ontological Semantic Model
TTC	Time-to-Collision
UTM	Universal Transverse Mercator
